# Probing the Role of Accessory FeS Clusters in Putative Sensory Group D [FeFe] Hydrogenase: Influence of the C‑Terminal [4Fe-4S] Cluster on the Reactivity of *Tam*HydS

**DOI:** 10.1021/acs.biochem.5c00784

**Published:** 2026-04-01

**Authors:** Conrad Schumann, Maximilian Böhm, Prasenjit Bhowmik, Ping Huang, Princess R. Cabotaje, Henrik Land, Gustav Berggren

**Affiliations:** 530001 Uppsala University, Department of Chemistry—Ångström, Molecular Biomimetics, 751 20 Uppsala, Sweden

## Abstract

Hydrogenases are metalloenzymes that play key roles in H_2_ metabolism. Beyond their catalytic function, [FeFe] hydrogenases from phylogenetic groups C and D have been proposed to act as H_2_ sensors. These putative sensory enzymes contain, in addition to the canonical H_2_-activating H-cluster, N-terminal [4Fe-4S] clusters as well as an atypical C-terminal [4Fe-4S] cluster, the latter being further associated with a Per–Arnt–Sim (PAS) domain in group C enzymes. The functional significance of these C-terminal clusters and their influence on enzymatic activity, however, remain poorly understood. Here we studied the accessory [4Fe-4S] clusters in the model group D enzyme from *Thermoanaerobacter mathranii* (*Tam*HydS), by disrupting cluster formation in the N- and C-terminal domains, respectively. Spectroscopic and biochemical investigations indicated that one of the N-terminal [4Fe-4S] clusters is critical for the structural integrity of *Tam*HydS. In contrast, disrupting the C-terminal [4Fe-4S] cluster through a cysteine to alanine mutation (C379A) resulted in a stable enzyme variant with a modified cluster. The C379A variant retained catalytic activity similar to the WT enzyme, although with a 2-fold enhancement of the H_2_ oxidation rate observed under high-driving-force conditions. Based on electron paramagnetic resonance spectroscopy, we found the oxidation state of the C-terminal cluster to be unresponsive to the presence of H_2_ gas. We inferred that the H_2_-sensing or signaling function is unlikely to involve redox state changes in the C-terminal [4Fe-4S] cluster. Still, highly conserved charged residues around the [4Fe-4S] clusters of group D [FeFe] hydrogenases indicate a functional role of the C-terminal region.

## Graphical abstract

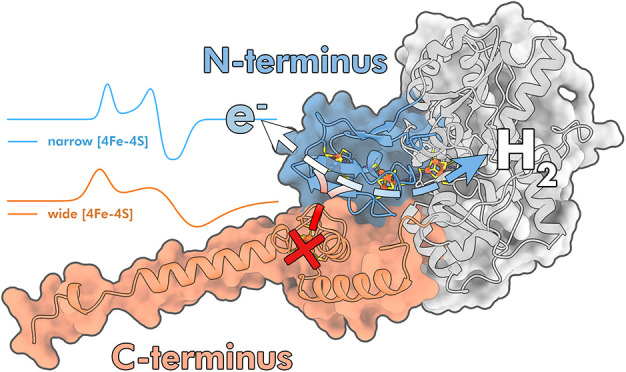

## Introduction

Iron–sulfur (FeS) clusters, with [2Fe-2S], [3Fe-4S], and [4Fe-4S] being the most common types, are ubiquitous inorganic cofactors that are essential for electron transfer (ET) in many fundamental biological processes like photosynthesis, respiration, and reduction–oxidation enzyme catalysis.^1,2^ In protein structures, the iron ions are typically coordinated by cysteine residues, although alternative FeS cluster ligands have been reported tuning the reduction potential of the cluster for specific functions.^3^ Beyond electron transfer, the integrity of these clusters is also crucial for the function of several proteins involved in DNA damage signaling, repair and replication, as well as catalysis.^4–9^ The diverse reduction and coordination properties of FeS clusters also enable certain FeS proteins to act as sensors for gases (O_2_, NO) or oxidative stress, regulating protein conformation or gene expression in response.^2,10–13^

The active sites of [NiFe] and [FeFe] hydrogenases are generally deeply buried in the protein, and they rely on the presence of FeS clusters for the intra- and intermolecular ET for the enzymatic interconversion of H_2_ to protons and electrons.^14^ [FeFe] hydrogenases have been shown to have the highest turnover frequencies with up to 10^4^ s^–1^ which is why they are of particular interest for energy conversion or biocatalytic applications.^15–20^ Even though this enzyme family is extremely diverse, with seven phylogenetically distinct groups identified to-date (groups A-G, Figure 1), all characterized [FeFe] hydrogenases share the same active site cofactor.^21–23^ The active site, the H-cluster, is composed of a di-iron subsite ([2Fe]_H_) connected via a cysteine to a cuboidal [4Fe-4S] cluster ([4Fe-4S]_H_). The di-iron site is further coordinated by a bridging azadithiolate (^−^SCH_2_NHCH_2_S^–^, ADT) along with CO and CN^–^ ligands.^24–28^ Between the phylogenetic groups, the electrochemical behavior of the H-cluster can vary as a result of different second- and outer-coordination sphere effects, which stabilize or destabilize catalytic intermediates. These stabilizing properties can tune the catalytic bias, affect the reversibility of the H_2_ turnover, and facilitate or prevent the formation of inhibited states.^29–33^ For the putatively sensory [FeFe] hydrogenases *Thermotoga maritima* HydS (*Tm*HydS, group C) and *Thermoanaerobacter mathranii* HydS (*Tam*HydS, group D), the stabilization of one-electron reduced H-cluster intermediates, H_red_ and H_red_H^+^, over a wider range of potentials has been argued to induce irreversible electrochemical behavior, in contrast to the reversible catalytic behavior observed for “prototypical” (group A) [FeFe] hydrogenases.^34–36^

**Figure 1:**
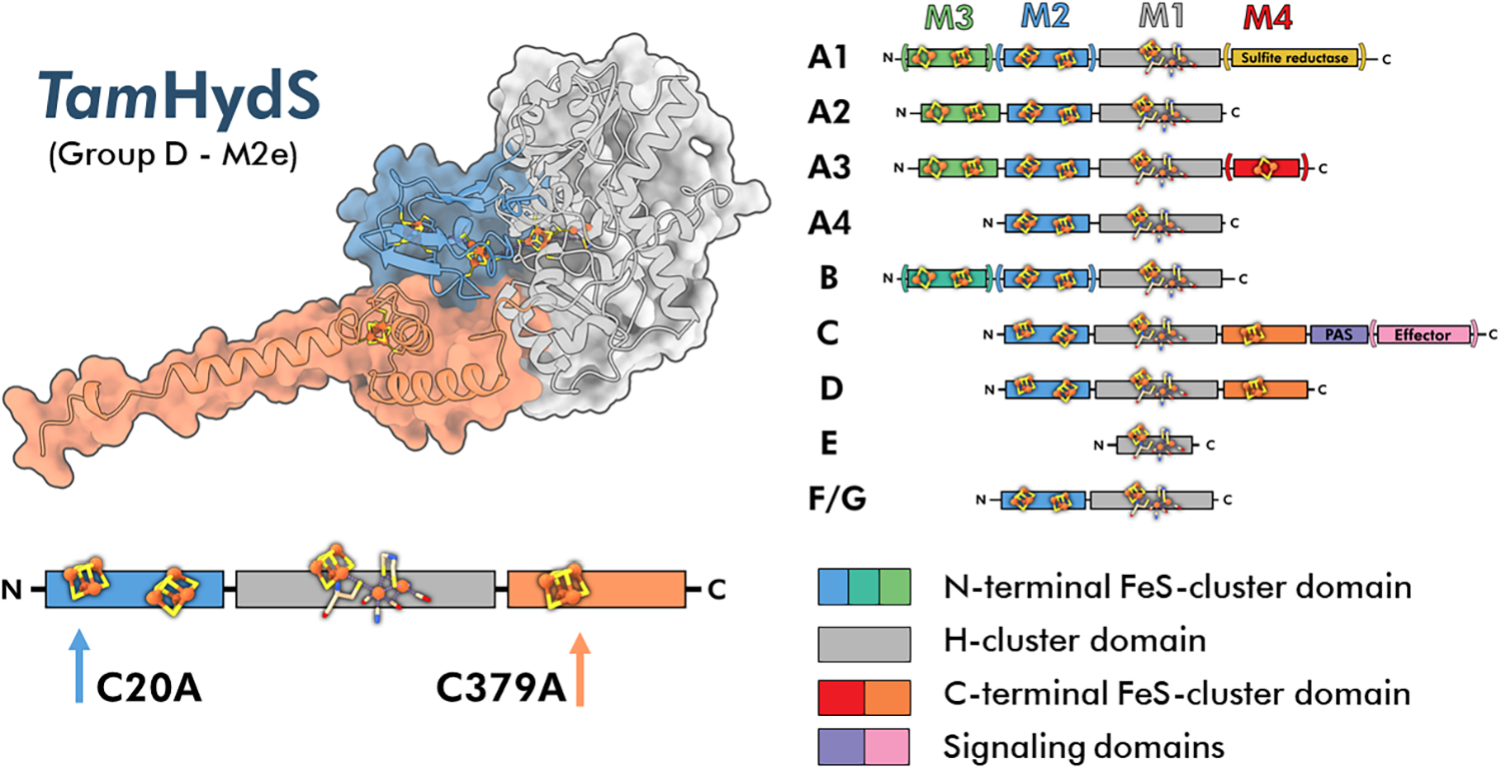
Left panel: AlphaFold2 predicted structure of a *Tam*HydS monomer, shown as a cartoon within a surface representation including three accessory [4Fe-4S] clusters and the active site consisting of the [4Fe-4S]_H_ and the di-iron subsite. Domains are colored as follows: N-terminal F-domain (blue), H-domain (gray), and C-terminal domain (orange). A linear domain organization scheme below the structure uses the same color scheme and highlights the locations of amino acid exchanges for the *Tam*HydS variants in this study. Right panel: Classification of [FeFe] hydrogenase catalytic subunits into phylogenetic groups (A–G) showing the domain organization of N- or C-terminal domains relative to the H-domain. [FeFe] hydrogenases are further categorized based on the presence and count of additional domains. A parentheses around a domain indicates the existence of individual enzymes with and without that specific additional domain within a given group.

In addition to the H-cluster, [FeFe] hydrogenase turnover depends on the kinetics of long-range proton and electron transfer to and from the active site. Proton transfer is mediated by amino acid residues of the catalytic domain (H-domain), which appear to be mainly conserved within the phylogenetic groups but can differ between groups.^37–39^ Intramolecular ET is instead primarily facilitated through relay FeS clusters in accessory domains (F-domains), which exist across most phylogenetic groups, with some exceptions in groups A and E.^22^ [FeFe] hydrogenases are further classified into subclasses (M1–M5), based on the domain count and architecture of the catalytic subunit.^40^ Investigating the F-domains is essential for understanding the electron transfer properties of the FeS clusters, their influence on the catalytic bias of H_2_ turnover, and the broader impact on hydrogenase properties. Previous studies have focused on probing the influence of the more commonly found accessory N-terminal FeS clusters, in the group A [FeFe] hydrogenases *Clostridium acetobutilycum* (*Ca*HydA) and *Megasphaera elsdenii* (*Me*HydA).^41–45^ The role of the C-terminal [4Fe-4S] cluster harboring domain, which appears to be a unifying structural feature in putatively sensory [FeFe] hydrogenases from phylogenetic group C (M2f) and group D (M2e) remains unexplored. For *Tm*HydS (group C) and *Tam*HydS (group D), the C-terminal [4Fe-4S] cluster’s involvement in sensing and/or signal transduction has been proposed independently, but not tested.^35,36^ While both characterized enzymes possess the C-terminal [4Fe-4S] cluster, only *Tm*HydS contains downstream of the [4Fe-4S] cluster an additional PAS-domain, which is typically associated with intracellular sensing and signal transductions.^46,47^ For *Tam*HydS on the other hand, the reaction with H_2_ has been reported to induce secondary structure changes, which could be important for a possible signaling mechanism.^48^

In the present study, we have investigated the role of two accessory [4Fe-4S] clusters of the assumed sensory [FeFe] hydrogenase *Tam*HydS from group D. The cysteine residues C20 and C379, coordinating one of the N-terminal [4Fe-4S] clusters and the C-terminal [4Fe-4S] cluster, respectively, were exchanged for alanine generating the two *Tam*HydS variants “C20A” and “C379A” (Figure 1). X-band electron paramagnetic resonance (EPR) spectroscopy revealed a decreased cofactor stability for C20A, whereas a [3Fe-4S] cluster was integrated into the C-terminal site for C379A. The spectroscopic and catalytic characteristics of the functional variant C379A were further analyzed using EPR spectroscopy, biochemical assays, and protein film voltammetry (PFV), and were compared to the wild-type (WT) enzyme. The C379A variant displayed catalytic properties similar to those of the WT enzyme, although the introduced [3Fe-4S] cluster in the C379A variant resulted in a modest enhancement of the H_2_ oxidation activity at a high driving force and low pH. We show that the C-terminal [4Fe-4S] cluster of *Tam*HydS is not reduced upon H_2_ exposure, which undermines the hypothesis that the C-terminal cluster’s redox state is involved in the H_2_ signaling. The observations are interpreted in the context of the predicted protein structure of *Tam*HydS and the electrostatic protein environment of the FeS clusters, considering also the sequence conservation within hydrogenase phylogenetic group D.

## Materials and Methods

### General

All chemicals were procured from Sigma-Aldrich, VWR, or Fisher Scientific unless otherwise stated. Protein expression and purification were analyzed by SDS-PAGE. The protein concentrations and Fe/protein contents were determined after each step using a Pierce Bradford Protein Assay Kit (Thermo Scientific) and a reported ferrous iron quantification assay.^49,50^ All sample preparation and activity analyses were conducted within an MBRAUN glovebox, maintaining oxygen levels below 10 ppm. The synthetic cofactor [Fe_2_(μ-ADT)­(CO)_4_(CN)_2_]­([Et_4_N]_2_) ([2Fe]^ADT^) was synthesized according to established literature protocols, with minor modifications on the scale of the synthesis, and its identity was confirmed by ATR-FTIR spectroscopy.^24,51^ UV/vis spectra were acquired using a TECAN Infinite M Nano UV/vis spectrometer and an AvaSpec-ULS2048-USB2-UA-50 Avantes fiber-optic UV/vis/NIR spectrometer.

### Site-Directed Mutagenesis

The gene encoding *Tam*HydS (NCBI: WP_418365830.1) with a C-terminal Strep-tag II was synthesized and cloned into pET-11a­(+) by GenScript using restriction sites NdeI and BamHI following codon optimization for expression in *Escherichia coli* (Table S1). Isolation of a pET-11a­(+) plasmid was performed with the GenElute Plasmid Miniprep Kit (Sigma-Aldrich). The site-directed mutagenesis PCR was performed using mutation primers (Table S1, Eurofins Genomics) with Phusion High-Fidelity DNA polymerase (Thermo Fisher Scientific) according to the manufacturer’s instructions with varying amounts of DMSO and an annealing temperature of 64 °C for optimal amplification conditions. Subsequently, template DNA was removed by incubation with FastDigest DpnI (Thermo Fisher Scientific) for 2 h at 37 °C, followed by 5 min at 80 °C, before the purity was evaluated on a 1% agarose gel. The linear PCR product was used to transform chemically competent *E. coli* BL21 (DE3) cells via heat shock at 42 °C. The circularized plasmids were extracted, and the sequence was confirmed via Sanger sequencing (Eurofins Genomics).

### Enzyme Preparation

The preparation of wild-type *Tam*HydS and two variants (C20A and C379A) in their respective apo-forms was performed following protocols previously reported for WT *Tam*HydS with minor changes to the procedure.^36^ For the expression of the apo-form, sequence-confirmed plasmids were transformed into chemically competent *E. coli* BL21­(DE3) cells. The cells were grown aerobically at 37 °C in M9 minimal medium (48 mM Na_2_HPO_4_, 22 mM KH_2_PO_4_, 9 mM NaCl, 18 mM NH_4_Cl, 4 g L^–1^ d-glucose, 2 mM MgSO_4_, and 0.1 mM CaCl_2_), supplemented with ampicillin at a final concentration of 100 μg mL^–1^. After reaching an OD_600_ ≈ 0.6, the cultures were supplemented with FeSO_4_ in 1% HCl solution (100 μM final concentration), before inducing the protein expression with 0.5 mM IPTG. Induced cultures were cultivated aerobically at 20 °C for approximately 18 h and subsequently harvested by centrifugation. The cell lysis, protein purification, reconstitution of the [4Fe-4S] clusters, as well as activation of the enzyme, were carried out in an MBRAUN glovebox under an argon atmosphere. The harvested cell pellets were lysed by sonication in 100 mM Tris–HCl, 150 mM NaCl, pH 8.0, supplemented with 1 g L^–1^ lysozyme, 0.05 g L^–1^ DNase, 0.05 g L^–1^ RNase, 2 g L^–1^ MgCl_2_·6 H_2_O, cOmplete Protease Inhibitor Cocktail (Roche), 5 g L^–1^ sodium deoxycholate, 50 g L^–1^ sucrose, 2 mM DTT, and 0.1% Triton-X100. The protein was purified using StrepTrap affinity chromatography (StrepTrap XT (Cytiva)) following the manufacturer’s instructions and applying an additional washing step with 5 mM ATP and 10 mM MgCl_2_ in 100 mM Tris–HCl, 150 mM NaCl at pH 8.0, for the removal of contaminant Hsp70 molecular chaperones.^52,53^ An additional size-exclusion chromatography step (Superdex 200 Increase (Cytiva)) was carried out for the removal of small molecular weight impurities, using 100 mM Tris–HCl, 150 mM NaCl, pH 8.0 as elution buffer. Based on the chromatogram and an SDS-Page analysis, fractions with the target protein were pooled and concentrated using centrifugal filters (Amicon Ultra Centrifugal Filter, 30 kDa MWCO (Millipore)). To fully reconstitute the intact [4Fe-4S] cluster sites, the isolated enzymes were then incubated for 3 h with 50 μM enzyme, 500 μM dithiothreitol, 500 nM cysteine desulferase (CsdA) from *E. coli*,^54^ 700 μM l-cysteine, and 700 μM (NH_4_)_2_Fe­(SO_4_)_2_(H_2_O)_6_ in 100 mM Tris–HCl, 150 mM NaCl, pH 8.0.

Subsequently, the apo-forms of the WT and C379A variant were treated with the active site mimic [2Fe]^ADT^ to prepare the holo-form of each enzyme. The activation reaction containing 50 μM enzyme, 1 mM sodium dithionite, and 600 μM [2Fe]^ADT^ in 100 mM phosphate buffer, pH 6.8, was incubated for 2 h at 25 °C, and subsequently desalted using a PD-10 column and 10 mM Tris–HCl, pH 8.0. The successful activation and the correct cofactor integration were verified by ATR-FTIR and X-band EPR analyses. The synthetic activation of C20A did not result in the integration of the [2Fe]_H_ subsite; despite repeated efforts, no holoenzyme could be generated through this method. The generated holo-forms of [2Fe]^ADT^-activated WT and C379A were concentrated to 510 ± 70 and 510 ± 44 μM, respectively. Aliquots were prepared in airtight vials and flash frozen in liquid N_2_ and stored at −80 °C.

### Continuous Wave Electron Paramagnetic Resonance (CW-EPR) Spectroscopy

All CW-EPR experiments were performed at the X-band frequency and at cryogenic temperatures (10–21 K) using a Bruker ELEXYS E500 spectrometer with a SuperX EPR049 microwave bridge and a cylindrical TE_011_ ER 4122SHQE cavity cooled via an Oxford Instruments continuous flow cryostat. Temperatures during the experiments were controlled and monitored with an Oxford Instruments ITC 503 temperature controller. For the data acquisition and primary processing of the spectra, Xepr software (Bruker) was used. All spectra were normalized to a microwave frequency of 9.4 GHz before being plotted with Origin 2019 (OriginLab Corporation). Further spectral simulations and fitting of spectral components were performed using the EasySpin software, version 6.0.8.^55^

### H_2_ Evolution Assay

The activated holoenzyme variants were tested for hydrogen evolution activity using a previously reported method with minor adjustments for a stopped reaction assay. 400 μL enzyme solution (2 μM) was prepared in sodium phosphate buffer (100 mM, pH 6.8) and sealed with a butyl rubber septum in a 9 mL GC-vial (PerkinElmer) in an MBRAUN glovebox under anaerobic conditions. Similarly, a solution of 20 mM methyl viologen (MV, *E* ^0′^ = −0.450 V vs SHE) and 200 mM sodium dithionite (NaDT) was prepared under the same conditions.^56,57^ The reaction was initiated by rapidly adding 400 μL of the MV-NaDT solution to the enzyme solution resulting in final reaction mix containing 1 μM enzyme, 10 mM MV, and 100 mM NaDT. The reaction was then incubated with shaking for 15 min at 30 °C and subsequently stopped by the addition of 200 μL of 2 M NaOH. The produced H_2_ gas was then quantified using a modified PerkinElmer Clarus 590 gas chromatograph (Armel Engineered Solutions) equipped with a HayeSep N column (NR021501, PerkinElmer) and molecular sieve X13 column (NR022501, PerkinElmer) and a thermal conductivity detector (TCD). Samples were injected via a 1 mL sample loop using a PerkinElmer TurboMatrix 40 headspace autosampler with argon as the carrier gas. For each enzyme variant, at least five technical replicates were analyzed to determine the specific activity (U mg^–1^). One unit corresponds to the production of 1 μmol of H_2_ per minute under the specific conditions of the assay. A one-way ANOVA and a Tukey’s HSD test were performed to test for statistically significant differences between the variants.

### H_2_ Oxidation Assay

The H_2_ oxidation activity of the activated holoenzyme variants was tested in an MBRAUN glovebox under anaerobic conditions at 25 °C using previously reported methods with minor adjustments.^39,58^ Benzyl viologen (BV, *E* ^0′^ = −0.359 V vs SHE) and methylene blue (MB, *E* ^0′^ = 0.011 V vs SHE) were used as redox mediators and spectroscopic probes for the catalyzed reaction.^56,57,59^ Each reaction contained 50 nM enzyme in H_2_-saturated potassium phosphate buffer (100 mM, pH 6.8) and either 50 μM MB or 500 μM BV. The reaction was initiated by the rapid addition of the H_2_-saturated buffer into gastight quartz cuvettes containing the enzyme and the respective redox mediator. The H_2_ oxidation activities with low or high driving force were measured following the initial rate of the formation of BV_red_ at 550 nm (ε_red,550 nm_ = 9.12 mM^–1^ cm^–1^) or the depletion of MB_ox_ at 665 nm (ε_ox,660 nm_ = 37.9 mM^–1^ cm^–1^), respectively. One unit (U) corresponded to the formation of 2 μmol of BV_red_ and the depletion of 1 μmol of MB_ox_ per minute, respectively (equivalent to 1 μmol of H_2_ oxidized per min), under the specific conditions of the assay. All initial rates were measured in at least four technical replicates and plotted as the mean with standard deviation. To test for statistically significant differences between the variants, a one-way ANOVA and a Tukey’s HSD test were performed.

### Protein Film Voltammetry (PFV)

All PFV experiments were performed in an MBRAUN glovebox under anaerobic conditions at 25 °C. The three-electrode setup comprised an Ag/AgCl (4 M KCl) reference electrode, a graphite rod counter electrode, and a 5 mm OD edge-plane pyrolytic graphite rotating disk working electrode embedded into a PEEK body (15 mm OD). Before use, the working PGE electrode surface was roughened using P1200 sandpaper, sonicated for approximately 1 min, and rinsed with deionized water. The working electrode was rotated at 3000 rpm for all measurements. Before the preparation of the protein film, adsorbed oxygen was removed from the working electrode by performing cyclic voltammograms (CVs) at reducing potentials of 50 mV/s for 10 min. For the immobilization of the enzyme, an enzyme solution (5 μL of 5 μM in 10 mM HEPES buffer, pH 7) and polymyxin (5 μL of 0.2 mg mL^–1^ in water) were mixed on the electrode surface and left for 5–10 min to adsorb before the excess of the solution was removed with a pipet. Experiments were performed at 25 °C in a gastight glass cell with hydrogen and argon flow gas inlets/outlets as well as a water jacket for temperature control. CVs with the bare working electrode (blank) and immobilized enzyme were recorded at 5 mV s^–1^ in a potential window – 300 to 300 mV (vs RHE). The electrochemical buffer, containing 5 mM MES, 5 mM CHES, 5 mM HEPES, 5 mM TAPS, and 5 mM sodium acetate, and 100 mM Na_2_SO_4_ as the supporting electrolyte, was titrated to the desired pH using NaOH or H_2_SO_4_. The headspace and buffer in the cell were equilibrated for at least 10 min with varying partial pressures of H_2_ before each measurement. The pH study experiments were performed with stable films between pH 5.0 and 8.0 with pH 6.0 and 1 atm of H_2_ as standard conditions to assess the film stability throughout the experiments. Two protein film replicates of each enzyme were analyzed for the pH dependence of high-driving-force slopes. Electrochemical data was acquired and analyzed using the GPES (Metrohm/Autolab) and Origin 2016 software, respectively.

### *In Silico* Protein Structure and Sequence Analysis

The three-dimensional structure of *Tam*HydS was predicted as a homodimer and as a monomer using AlphaFold2.^60^ The predicted homodimer interface was further examined using the PDBePISA online tool (EMBL-EBI).^61^ The di-iron site and N-terminal FeS clusters were subsequently modeled into the predicted structure by structural superposition with the [FeFe] hydrogenase CpI from *Clostridium pasteurianum* (PDB: 4XDC) using ChimeraX.^62,63^ The C-terminal FeS cluster was manually positioned into its predicted binding site. The quality of the predicted protein structure was evaluated based on the predicted local distance difference test (pLDDT) and predicted aligned error (PAE) scores. Relative domain positioning was further scrutinized using the DeepAssembly deep learning algorithm, which predicts interdomain interactions.^64^ These predictions were then cross-compared to the AlphaFold2 model. Internal cavities, specifically the FeS cluster binding sites, were identified and visualized within the predicted protein structure using the ChimeraX-integrated tool KVfinder.^65^ Subsequently, the Coulombic electrostatic potential (ESP) was mapped onto these internal cavities using ChimeraX.^63^ Amino acid sequence analysis, including composition and domain-specific theoretical isoelectric points (pI), was performed using ProtParam on the Expasy Server.^66^ The sequence conservation was probed via a multiple sequence alignment (MSA) of 70 group D [FeFe] hydrogenase sequences using Clustal Omega (EMBL-EBI).^67^ The amino acid numbering in the Jalview-generated sequence logo follows the residue numbering of *Tam*HydS. Insertion of aligned sequences were removed and only highly conserved residues, with >75% residue identity, were colored.^68^

## Results

### Apoenzyme Preparation and EPR Characterization of Their FeS Clusters

The two *Tam*HydS variants C20A and C379A, as well as WT *Tam*HydS, were heterologously produced in *E. coli* BL21 cells, and isolated under strictly anaerobic conditions using affinity chromatography and subsequent size-exclusion chromatography (Figures S1, and S2). The isolated enzymes were expected to lack the [2Fe]_H_ subsite, as the H-cluster maturation enzymes HydEFG were not present in the employed *E. coli* strain.^69^ In the following, we will refer to preparations of the enzymes that lack the catalytic [2Fe]_H_ subsite as an apoenzyme, while the holoenzymes include the complete H-cluster. As the WT enzyme features four [4Fe-4S] cluster binding sites, an iron content of 16 Fe/protein is expected, while an iron content of 12–16 is expected for the two variants depending on the extent of Fe–S cluster assembly in the modified binding site.^36^ The purification of WT and C379A yielded apoenzymes with 8 ± 1 Fe/protein and 3 ± 1 Fe/protein, respectively, resulting in a need for postpurification *in vitro* reconstitution of the FeS clusters. Semienzymatic FeS cluster reconstitution, employing Mohr’s salt and cysteine desulferase (CsdA) and l-cysteine for generation of sulfide, resulted in an absorbance increase around 410 nm, indicating the integration of additional [4Fe-4S] clusters for both WT and C379A.^54^ However, the resulting UV/vis absorbance spectra revealed a lower extinction coefficient around 410 nm for C379A compared to that for WT and hence differences in the FeS cluster integration (Figure S3). Iron quantification assays further supported the notion of a lower Fe/protein content for the C379A variant after the reconstitution, with final Fe contents of 14 ± 3 Fe/protein and 11 ± 1 Fe/protein for WT and the C379A variant, respectively. These reconstituted forms of the proteins were used in all subsequent experiments. The C20A variant was isolated with 12 ± 1 Fe/protein and therefore regarded as fully iron loaded and already as-isolated (Table S2).

For a more detailed investigation of the FeS clusters in the apoenzymes, X-band EPR spectroscopy was performed. In line with earlier reports, NaDT reduction of the reconstituted apo-WT prepared for this study resulted in two rhombic spectral components which were confirmed through spectral simulations: a wider rhombic signal with *g*_1,2,3_ = 2.064, 1.886, and 1.839 and a narrower rhombic signal with *g*_1,2,3_ = 2.056, 1.935, and 1.890 (Figure 2A). Even though Fe quantification suggested a high Fe/protein content for apo-C20A, NaDT-treated samples resulted in no EPR-active species. Instead, the as-isolated (nonreduced) apo-C20A showed weak EPR signals at *g* = 4.31 and a signal centered around *g* = 2.02. These signals are characteristic for advantageously bound (or “free”) Fe^3+^ and [3Fe-4S]^+^ cluster species, indicating unspecific, labile iron binding or misfolding of the C20A variant (Figure S5). Conversely, the EPR spectrum of NaDT-treated reconstituted apo-C379A revealed the presence of a relatively narrow rhombic [4Fe-4S]^+^ cluster signal compared to apo-WT (Figure 2B). Simulations of the spectra of reduced apo-C379A returned good fits with a single spectral component (*g*_1,2,3_ = 2.057, 1.937, and 1.885). Additionally, comparing EPR samples of as-isolated apo-C379A and apo-WT prepared in the absence of dithionite revealed a weakly anisotropic EPR signal (*g*_1,2,3_ = 2.035, 2.020, and 1.973) in both enzymes (Figure 2C and S5C). However, the signal was 3-fold larger in C379A relative to the WT enzyme. As noted above, the *g* = 2.02 centered signal is typically associated with [3Fe-4S]^+^ clusters, which are EPR active in their oxidized state but become EPR silent upon reduction.

**Figure 2:**
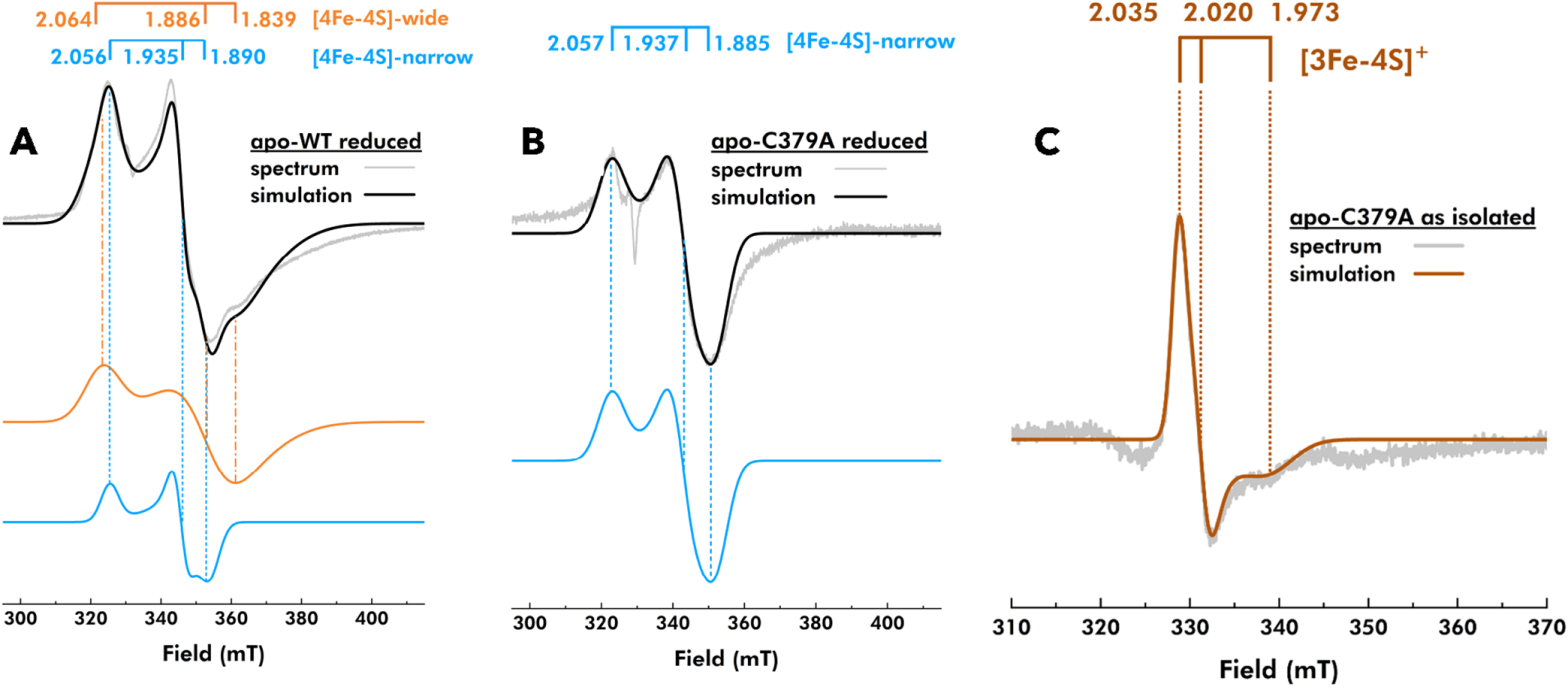
Analysis of apo-WT and apo-C379A by X-band EPR spectroscopy. (A) EPR spectrum of NaDT-reduced apo-WT enzyme (gray spectrum) was simulated with two spectral components, a [4Fe-4S]-wide with *g*_1,2,3_ = 2.064, 1.886, and 1.839 (orange) and a [4Fe-4S]-narrow with *g*_1,2,3_ = 2.056, 1.935, and 1.890 (blue). (B) EPR spectrum of NaDT-reduced apo-C379A (gray spectrum) required only one spectral component which resembled [4Fe-4S]-narrow from apo-WT with similar *g*-values *g*_1,2,3_ = 2.057, 1.937, and 1.885 (blue). (C) EPR spectrum of as-isolated (nonreduced) apo-C379A (gray spectrum) showed a narrow rhombic signal which was simulated with *g*-values *g*_1,2,3_ = 2.035, 2.020, and 1.973 (orange), characteristic for a [3Fe-4S]^+^ cluster. NaDT-reduced apoenzyme samples contained 50 μM enzyme and 1 mM NaDT in 100 mM Tris–HCl at pH 8.0. X-band EPR spectra were collected at 10 K and 80 μW microwave power with a microwave frequency of 9.4 GHz, a modulation frequency of 100 kHz, and a modulation amplitude of 1 mT. Spectral simulations were performed using the software EasySpin version 6.0.8.^55^

The combined EPR data collected on the reduced and nonreduced samples of both C379A and WT allow us to assign the two rhombic components of the WT, with the “wide” signal (*g*_1,2,3_ = 2.064, 1.886, 1.839) arising from the reduced C-terminal cluster and the “narrow” signal (*g*_1,2,3_ = 2.057, 1.937, 1.885) attributable to one of the two [4Fe-4S] clusters of the N-terminal domain. This assignment is supported by the fact that the “narrow” rhombic EPR signal is observed in both WT and C379A under reducing conditions, while the “wide” component is evident only in the WT enzyme. Further, loss of the “wide” rhombic signal in combination with the increased magnitude of the *g* = 2.02 signal in C379A indicates that the modified C-terminal FeS cluster binding site has lost the capacity to bind a [4Fe-4S] cluster and, instead, partially incorporates a [3Fe-4S] cluster.

### Preparation of the Holoenzymes and EPR Studies of Their Reactivity toward H_2_

The pure holoenzymes of *Tam*HydS-WT and the C379A variant were generated by mixing the apoenzymes with the synthetic [2Fe]_H_ subsite precursor [2Fe]^ADT^, which resulted in the for *Tam*HydS characteristic H-cluster signatures in FTIR spectroscopy (Figure S4).^25,36^ For the C20A variant, on the other hand, similar treatments consistently failed to generate the holoenzyme, presumably due to the aforementioned insufficient protein integrity or impaired FeS cluster integration. Spectroscopic analysis via X-band EPR of holo-WT and holo-C379A in the isolated state confirmed the assembly of the H-cluster. Samples of holo-WT, flash frozen in pH 8 buffer without further treatment, revealed two rhombic components, denoted R1 (*g*_1,2,3_= 2.099, 2.044, 2.010) and R2 (*g*_1,2,3_= 2.109, 2.053, 2.010), characteristic for *Tam*HydS in the catalytic resting state H_ox_ (Figure 3A). The same two rhombic features were evident in spectra collected on C379A prepared under identical conditions. The slight shifts in the *g*-values of holo-C379A in comparison to holo-WT are attributable to minor changes in structure and/or magnetic environment of the H-cluster between the enzymes. Although the spectrum of C379A is dominated by these two rhombic components, two additional minor features were also discernible. Through variation of the temperature and microwave power, the faster relaxing underlying spectral components were separated from the slower relaxing, H_ox_-associated components (Figure S6). A comparison with the apo-C379A spectrum illustrated the presence of a [3Fe-4S]^+^ cluster, as well as low levels of a fast-relaxing isotropic signal (*g* = 2.016) of unknown origin (Figures 3A, and S6).

**Figure 3:**
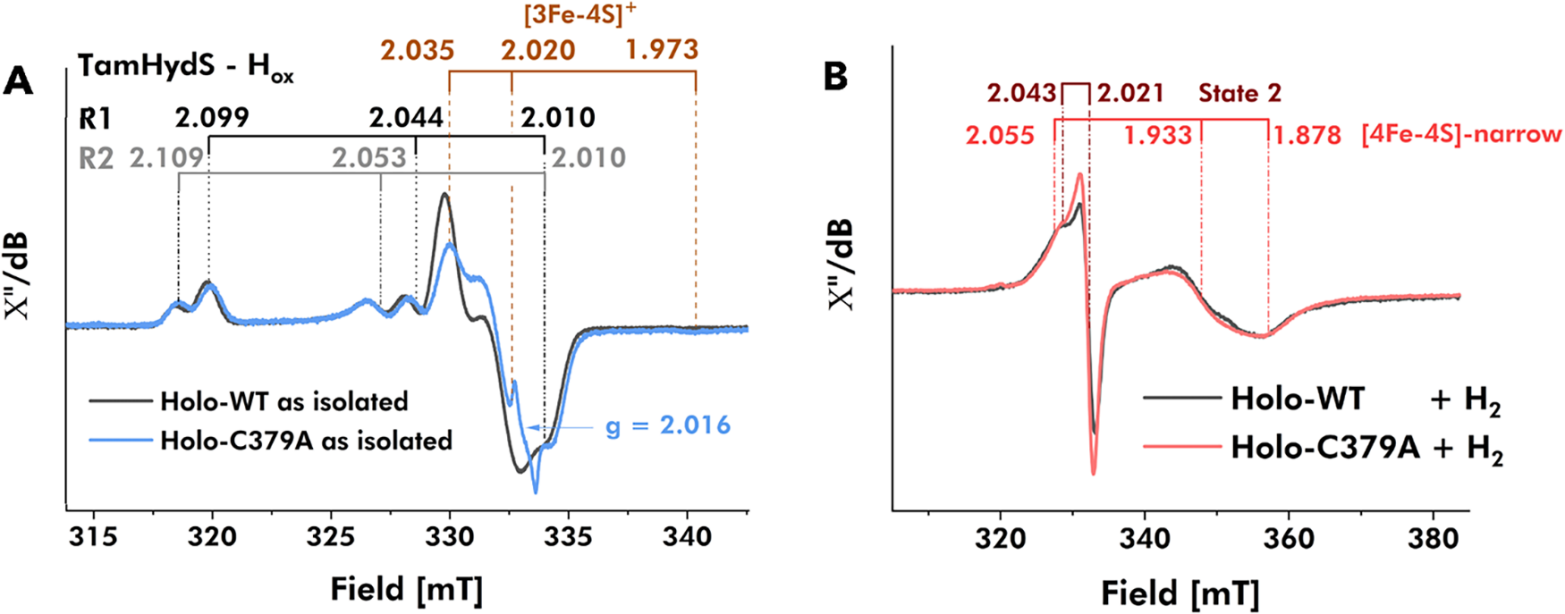
Analysis of holo-WT and C379A by X-band EPR spectroscopy. (A) EPR spectra collected of as-isolated forms of holo-WT (gray spectrum) and holo-C379A (light blue spectrum) show two major spectral components attributable to the H_ox_-state, R1 (*g*_1,2,3_ = 2.099, 2.044, 2.010) and R2 (*g*_1,2,3_ = 2.109, 2.053, 2.010). Holo-C379A contains additional spectral components attributed to a [3Fe-4S]^+^ cluster with *g*_1,2,3_ = 2.035, 2.020, and 1.973 and anisotropic signal with *g*_iso_ = 2.016 (note: spectra normalized at 320 mT). (B) Normalized EPR spectra of H_2_-reduced holo-WT (gray spectrum) and holo-C379A (red spectrum), both with two spectral components attributable to State 2 (*g*_**||**_ = 2.043, *g*_**⊥**_ = 2.021) and [4Fe-4S]-narrow (*g*_1,2,3_ = 2.055, 1.933, 1.878). All holoenzyme samples contained 50 μM enzyme in 100 mM Tris–HCl at pH 8.0, while H_2_-reduced samples were additionally incubated under 1 atm H_2_ for 30 min. X-band EPR spectra were collected at (A) 21 K and 16 μW and (B) 10 K and 80 μW microwave power with a microwave frequency of 9.4 GHz, modulation frequency of 100 kHz and a modulation amplitude of 1 mT.

Equivalent samples of WT and the C379A variant were then incubated under a H_2_ atmosphere aiming to (re)­investigate redox changes of the FeS clusters in response to H_2_ as the natural reductant. In line with previous studies of *Tam*HydS, incubating the holo-WT enzyme under 1 atm H_2_ in pH 8 buffer gave rise to a pseudoaxial signal, denoted “State 2”, and a rhombic signal which shares similarity with the narrow [4Fe-4S] cluster signal mentioned above.^39,58^ The analysis of H_2_-treated holo-WT samples at different pH values (pH 6 and 7) showed some variation in relative signal intensities, implying comparable EPR-active species in different ratios (Figure S8). Similarly, the H_2_-treated holo-C379A sample at pH 8.0 displayed spectra with the two spectral components in changed ratios compared to holo-WT (Figure 3B). Simulations of H_2_-reduced holo-C379A spectra confirmed that a good fit can be obtained with the two spectral components: State 2 (*g*_**||**_ = 2.043, *g*_**⊥**_ = 2.021) and [4Fe-4S]-narrow (*g*_1,2,3_ = 2.055, 1.933, and 1.878) (Figure S7). The absence of the [3Fe-4S]^+^ cluster and the fast-relaxing isotropic (“radical like”) signal in samples of H_2_-treated holo-C379A, suggests their reduction into EPR-inactive states after incubation with H_2_. However, a minor underlying spectral component of [3Fe-4S]^+^ cannot be completely ruled out with certainty, due to the overlapping signals of State 2 and [3Fe-4S]^+^. Critically, we note that the “wide” rhombic EPR signal, assigned to the C-terminal [4Fe-4S] cluster, is absent in spectra collected of both WT and C379A. This observation strongly argues against a reduction of this cluster under 1 atm of H_2_.

### Catalytic Characteristics of *Tam*HydS C379A

The effect of the amino acid exchange on the catalytic properties of the *Tam*HydS variant C379A were investigated via solution assays and protein film voltammetry (PFV). Solution assays for H_2_ evolution (H^+^ reduction) were performed at 30 °C and pH 6.8 using NaDT-reduced methyl viologen (MV, *E* ^0′^ = −0.450 V vs SHE) as the electron donor. All H_2_ oxidation solution assays were performed at 25 °C and pH 6.8 with either benzyl viologen (BV, *E* ^0′^ = −359 V vs SHE) or methylene blue (MB, *E* ^0′^ = 0.011 V vs SHE) as an electron acceptor. The H_2_ evolution solution assays revealed no statistically significant differences in the specific activities between WT and C379A. Similarly, there were no apparent differences when comparing the H_2_ oxidation activities in the low-driving-force assays with BV as an electron acceptor. However, C379A showed a 2-fold increase of specific activity for H_2_ oxidation as compared to the WT with MB as an electron acceptor (Figure 4, and Table S3). We tentatively attribute the enhanced H_2_ oxidation under high-driving-force conditions to the aforementioned integration of the [3Fe-4S] cluster.

**Figure 4:**
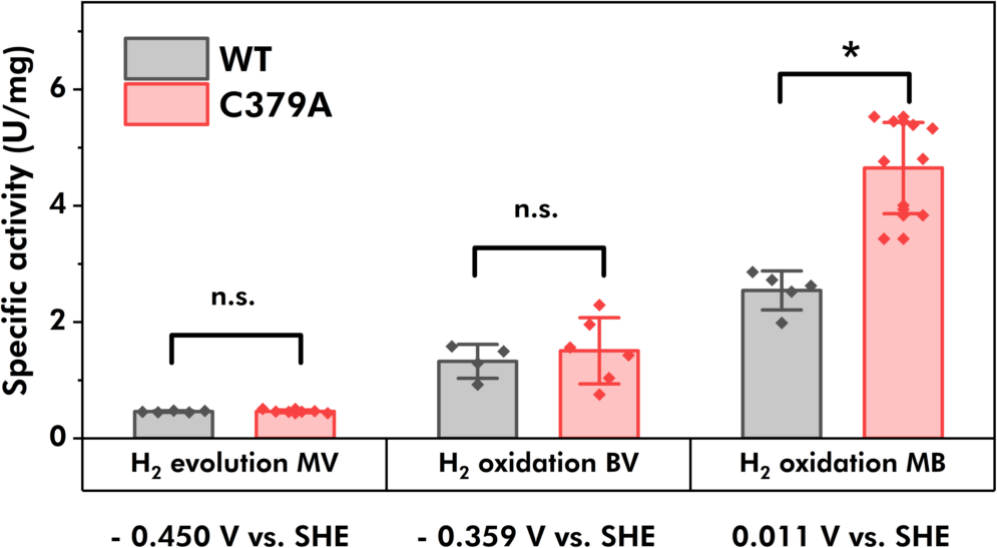
Comparison of *Tam*HydS (WT, gray bars) and variant C379A (red bars) regarding their specific activities for H_2_ evolution (H^+^ reduction) and H_2_ oxidation. Measured values are displayed as data points with the mean and standard deviation in the bars. H_2_ evolution activities were measured by gas chromatography at pH 6.8 and 30 °C using 10 mM methyl viologen (MV, *E* ^0′^ = −0.450 V vs SHE) as the redox mediator and 100 mM NaDT as the sacrificial electron donor. H_2_ oxidation activities were measured in colorimetric assays with 500 μM benzyl viologen (BV, *E* ^0′^ = −0.359 V vs SHE) or 50 μM methylene blue (MB, *E* ^0′^ = 0.011 V vs SHE) in H_2_-saturated phosphate buffer at 6.8 and 25 °C. One unit (U) is defined as the production or consumption of 1 μmol H_2_ min^–1^. The performed one-way ANOVA and a Tukey’s HSD test results are displayed as not significant (n.s.) and as significant difference between the means (*) with a significance level of <0.05.

The observed enhancement in H_2_ oxidation at high driving force was further examined employing PFV under different H_2_ partial pressures and various pH values (pH 5–8). The protein film was prepared on a highly oriented pyrolytic graphite electrode (PGE) using the polycation polymyxin B sulfate for enhanced film formation and stability.^36^ All cyclic voltammograms (CVs) were recorded at 25 °C; a slow scan rate (5 mV/s) was used to approach steady-state conditions, while the electrode was rotated at 3000 rpm to ensure efficient mass transport.

For both WT and C379A, currents for H^+^ reduction and H_2_ oxidation were evident together with the overpotential typical of *Tam*HydS (Figure 5, S9 and S10).^34,36^ The currents observed in PFV reflect both the turnover frequency of the enzyme and the surface loading of the enzyme, with the latter being an unknown quantity. Thus, to facilitate a comparison between WT and C379A, the CV traces with varied H_2_ partial pressures were normalized based on the current at the most reducing potential and then evaluated, while the CV traces with varying pH were analyzed without normalization. Additionally, as the currents do not reach a plateau, but instead continually increase with increased applied potential, the steady-state limiting current (related to *V*_max_) was estimated based on linear current increase (slope) at high driving force.^70^

**Figure 5:**
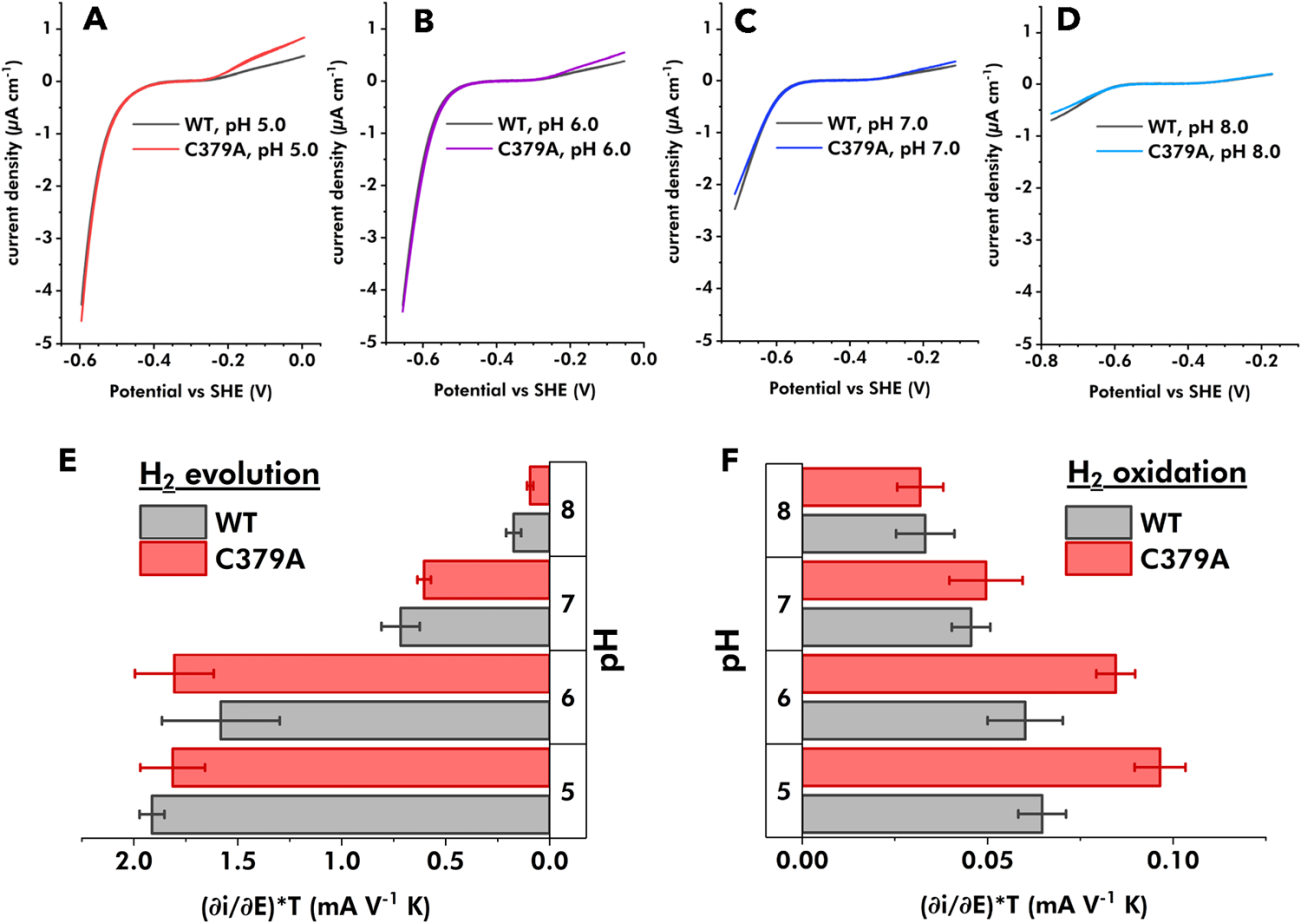
PFV analysis of *Tam*HydS variant C379A and WT. A–D: CVs of C379A (colored traces red to blue) and WT (black traces) in the pH range 5–8 were recorded with a scan rate of 5 mV s^–1^, an electrode rotation rate of 3000 rpm, under 1 atm H_2_, and at 25 °C. Enzymes were immobilized on individual rotating disc edge-plane pyrolytic graphite electrodes (PGE) in the presence of polymyxin B sulfate. CV traces shown for C379A and WT were acquired using a single film for each enzyme at different pH and confirming the film stability by returning to the initial conditions at pH 6.0 in between and after the experiments. The CVs’ slopes at high driving force were analyzed to estimate the steady-state limiting current for (E) the H_2_ evolution and (F) the H_2_ oxidation. The data is presented as the average and standard deviation (error bars) of slopes from two individual protein films for each enzyme.

The effect of the H_2_ concentration was studied by varying the H_2_ partial pressure in the headspace of the cell at a fixed pH of 6.0. The protein films of both C379A and WT showed, after normalization, a steady increase in the oxidation currents with increasing H_2_ partial pressure (Figure S9), in line with a *K*_M_ > 0.5 atm H_2_ for both enzymes, in agreement with earlier reports on WT.^34^ The recorded CV traces for C379A resulted in overall higher H_2_ oxidation limiting currents compared to the WT. This ratio of the observed limiting currents between variant C379A and WT was consistent over the entire tested range of H_2_ partial pressures (0.25–1 atm).

In parallel, the influence of pH on the overall current response was analyzed (Figures 5, and S10). The limiting currents (linear slope) at high driving force were here also used as a relative estimate for maximum catalytic rates (*V*_max_). WT and C379A showed comparable electrochemical behavior regarding the currents and overpotential under reducing conditions, implying similar catalytic capacity for H^+^ reduction across the entire tested pH range. An apparent pH optimum between pH 5 and pH 6 was observed for both WT and C379A, reflected in the highest limiting current for the H^+^ reduction, again in good agreement with earlier reports on WT.^36^

A similar pH trend was observed also on the oxidation side, with H_2_ oxidation currents increasing continuously with decreasing pH. This pH-dependent H_2_ oxidation activity increase was further enhanced in C379A, resulting in a nearly 2-fold higher relative limiting current at pH 5.0 compared to the WT. The combined results from the solution assays and the PFV indicate an enhancement of H_2_ oxidation activity that is independent of H_2_ partial pressure but dependent on both applied overpotential and pH for the *Tam*HydS variant C379A.

### Structure Prediction and Sequence Analysis

An initial inspection of the amino acid sequence of *Tam*HydS revealed an uneven distribution of negatively and positively charged amino acids between the N- and C-terminus of *Tam*HydS (see Table S4). To gain further insight into the FeS clusters of *Tam*HydS, the AlphaFold2^60^ (AF2) predicted protein structure was analyzed in the N- and C-terminal domains based on the amino acid sequence and the electrostatics in the proximity of the [4Fe-4S] clusters. Predictions of *Tam*HydS as a homodimer resulted in a parallel orientation of two protomers along the axis of the C-terminal helix (Figure S11). Conversely, an analysis of the interface between the two *Tam*HydS protomers using PDBePISA revealed a low probability for the interface to play a role in the formation of a complex (Table S5). Due to limited knowledge on the nature of a functional complex or protein–protein interactions of *Tam*HydS in the native host, only the monomer was considered in subsequent analyses. The AF2-structure prediction of the *Tam*HydS monomer resulted in a high structural confidence with a pLDDT of 94.17, which also is reflected in the mainly low PAE score. The PAE plot also shows that the prediction had high confidence in the relative domain position with the N- and C-terminal domain neighboring each other (Figure S12). The relative domain positions were also investigated using DeepAssembly,^64^ a deep learning algorithm for predicting interdomain interactions. The virtually identical AF2- and DeepAssembly modeled structures further supported the formation of salt bridges and hydrogen-bonds between the N- and C-terminal domains, which brings the C-terminal [4Fe-4S] cluster close to the N-terminal [4Fe-4S]. In the case of *Tam*HydS, these predicted interactions appear to involve R30, *R*32, and R42 and S384, E392, and D393 in the N- and C-terminal domain, respectively. R30, *R*32, E392, and D393 are also highly conserved (>75% residue identity) across 70 group D [FeFe] hydrogenases (Table S6), which suggest a structural or functional importance of the interaction within the phylogenetic group (Figures 6E,F, and S13).

**Figure 6:**
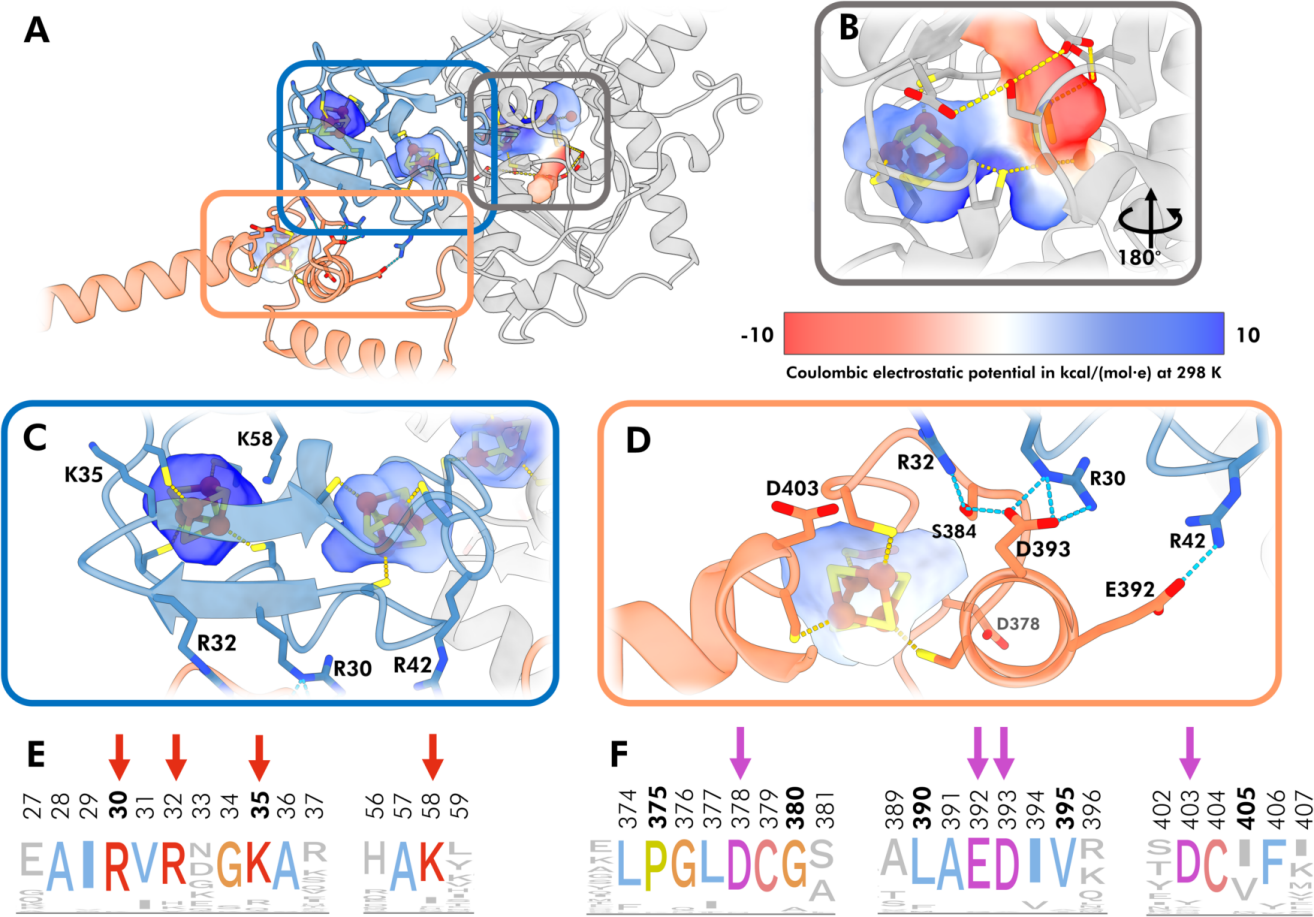
*In silico* analysis of electrostatic and sequence environment of FeS cluster binding sites in *Tam*HydS. (A) AlphaFold2 predicted structure of a *Tam*HydS monomer, shown as a cartoon with the Coulombic electrostatic potential mapped on internal protein surfaces around the FeS cluster binding sites excluding the electrostatic effects of the FeS cluster itself. Enlarged view of (B) the active site consisting of the [4Fe-4S]_H_ and the [2Fe]_H_ subsite (C) two N-terminal [4Fe-4S] cluster sites, and (D) one C-terminal [4Fe-4S] cluster. Domains are colored as follows: N-terminal F-domain (blue), H-domain (gray), and C-terminal domain (orange). Sequence logo showing conservation across 70 [FeFe] hydrogenases from the phylogenetic group D in parts of the (E) N-terminal and (F) C-terminal domain with colored residues corresponding to more than 75% residue identity in the consensus sequence. Positions highlighted with arrows are correspondingly labeled in the structural view above (C and D).

The *Tam*HydS AF2-structure was then further examined by mapping the electrostatic environment of the [4Fe-4S] and H-cluster binding sites, using pyKVfinder^65^ and ChimeraX^63^ as electrostatic charge around FeS clusters have been reported to affect their reduction potential.^71,72^ Around the H-cluster, the analysis revealed a positive electrostatic potential around the H-cluster cubane ([4Fe-4S]_H_) and a negative potential closer to the [2Fe]_H_ site (Figure 6B). The negative potentials close to the active site arise from the proton transport pathway featuring a series of negatively charged residues (E252, E289, S249, and D265, Figure 6D). Further, the analysis showed that the two N-terminal [4Fe-4S] clusters are located in more positively charged protein environments (Figure 6C), while the electrostatic potential around the C-terminal [4Fe-4S] cluster is comparatively neutral (Figure 6D). These differences in the electrostatic potential seem to result from the distinct amino acid compositions of the N- and C-terminal domains and the residues around the FeS cluster binding sites. In the C-terminal domain, the two aspartate residues D378 and D403 could have a substantial impact on the electrostatic environment as they are neighboring two of the cluster binding cysteines (C379 and C404). Conversely, the N-terminal domain of *Tam*HydS contains lysine residues (K58 and K35) with amine groups proximal to the N-terminal [4Fe-4S] clusters. Both, the aspartate residues in the C-terminal domain and the lysine residues in the N-terminal domain are also highly conserved (>75% residue identity) across 70 group D [FeFe] hydrogenases (Table S6), which supports a functional role these residues in group D [FeFe] hydrogenases (Figures 6 E, F, and S13).

## Discussion

In the present study, we applied site-directed mutagenesis to disrupt the formation of [4Fe-4S] clusters in *Tam*HydS by exchanging their coordinating cysteines, C20 and C379, with alanine. We found that altering these binding sites and by extension the structure and stability of the associated FeS cluster had a very different impact on the enzyme and cofactor integrity depending on the location of the cluster (N- or C-terminal). Targeting one of the N-terminal [4Fe-4S] clusters, in the C20A variant, resulted in large changes in overall cofactor assembly in the protein, as evident from the lack of [4Fe-4S]^+^ signals in the EPR spectra and our inability to generate a functional holoenzyme in the variant. We therefore conclude that the targeted N-terminal accessory [4Fe-4S] cluster not only serves the electron transfer but might also have a structural role in *Tam*HydS. Similar disruptive effects have been observed after site-directed mutagenesis of conserved cysteines involved in the [4Fe-4S] cluster coordination in, e.g., the nitrate reductase from *E. coli*.^5,73^

The C379A variant, on the other hand, resulted in the disruption of the C-terminal cluster, with no observable effects on the protein stability. We verified the modification of the C-terminal [4Fe-4S] cluster via UV/vis spectroscopy and iron quantification assays, which both showed a lower iron content in the C379A variant compared to that of the WT. EPR spectroscopy suggests that the C-terminal cluster has largely converted from a [4Fe-4S] cluster to a [3Fe-4S] cluster. We refrained from probing the occupancy of the binding site via absolute spin quantification, as the reduction potential of the [3Fe-4S] cluster and the spin coupling to the neighboring [4Fe-4S] clusters were not determined. We found, however, that the [3Fe-4S] cluster signal is observed with comparable intensity in both the apo- and the holoenzyme in two biological replicates of C379A, indicating structural homogeneity between replicates.

A comparison of EPR spectra collected on reduced apo-forms of WT and C379A allowed identification of the spectral signature of the C-terminal [4Fe-4S], as was the previously reported wide rhombic [4Fe-4S] component. Critically, this identification enabled us to probe redox state changes in the cofactors of the three separate domains (N-terminal domain, H-domain and C-terminal domain) upon H_2_ exposure. The H_2_ exposed holo-form of C379A resulted at pH 8.0 in the reduction of the H-cluster and one of the [4Fe-4S] clusters in the N-terminal domain, as well as the [3Fe-4S] cluster that was particularly present in holo-C379A. The reduction of the [3Fe-4S] cluster could be favored due to the more positive reduction potentials of [3Fe-4S] clusters in comparison to the [4Fe-4S] clusters. EPR spectra recorded on samples of the holo-forms of WT under 1 atm H_2_ in the pH range 6 to 8 were highly similar to those of C379A. Consequently, the C-terminal [4Fe-4S] cluster of the WT is not reduced under these conditions. This result complements our previous work on *Tam*HydS, which showed that photoreduction with Eosin Y and TEOA results in the reduction of two distinct [4Fe-4S] clusters in the holoenzyme, which we have now identified as one N- and the C-terminal [4Fe-4S] clusters of *Tam*HydS.^74^ Furthermore, we reported that both photoreduction and H_2_ incubation induce a secondary structure change at pH 8.0.^48^ Hence, the secondary structure change in the proposed H_2_ signaling mechanism evidently does not depend on the redox state of the C-terminal cluster.

The amino acid composition and the AF2-modeled structure of *Tam*HydS provide a possible rationale for the different redox behavior of the N- and C-terminal FeS clusters, namely, the different electrostatic environments of the FeS clusters. Charged residues surrounding the FeS clusters in *Tam*HydS are well conserved within the phylogenetic group D. More negatively charged residues (Asp and Glu) are located around the C-terminal [4Fe-4S] cluster, whereas the N-terminal [4Fe-4S] clusters are surrounded by more positively charged residues. The aspartates (D378 and D403) in the direct vicinity of the C-terminal cluster could act as point charges that tune the reduction potential of the C-terminal [4Fe-4S] cluster to a more negative value.^72,75^ This might explain why only the N-terminal cluster is reduced upon H_2_ exposure in the physiological pH range, while the reduction of the C-terminal cluster requires stronger reductive driving force as in the photoreduction with Eosin Y and TEOA.^74^

The amino acid substitution C379A in the C-terminal domain had only minor effects on the H_2_-turnover and the H_2_-induced active site state of *Tam*HydS, suggesting only a weak electronic interaction of the C-terminal FeS cluster with the active site. We could see no clear impact comparing the H_2_-production rates of WT and C379A. Yet, we found a pH-dependent increase in the H_2_ oxidation activity at high driving force in the C379A variant, resulting in a 2-fold higher H_2_ oxidation activity (pH 5) compared to the WT. We tentatively attribute this improvement in H_2_ oxidation rates to the [3Fe-4S] cluster, observed in holo-C379A. While the native C-terminal [4Fe-4S] is challenging to reduce, converting this cluster to a [3Fe-4S] species should make its reduction more facile by shifting the reduction potential to a more oxidizing potential.^75^ Thus, the [3Fe-4S] cluster could generate an additional electron sink for electron transfer from the H-cluster during catalysis. The involvement of an intermediate with a more positive reduction potential would also rationalize the observation that H_2_ oxidation rates are primarily enhanced at high-driving-force conditions.

## Conclusion

In conclusion, this study broadens the understanding of the structure–function relationship in the, to date, only characterized [FeFe] hydrogenases from the phylogenetic group D, and it challenges earlier hypotheses on possible signal transduction mechanisms. Additionally, this study provides another example of how [4Fe-4S] cluster binding sites can be engineered to bind functional [3Fe-4S] clusters.^75–77^

Our data show that the C-terminal cluster has a negligible effect on the catalytic properties, suggesting that the N-terminal domain also provides the primary electron transfer pathway in this group of hydrogenases. More surprisingly, the C-terminal cluster was not reduced even under 1 atm H_2_; thus, the redox state of the C-terminal [4Fe-4S] cluster is not associated with the secondary structure changes previously reported for *Tam*HydS as a potential H_2_ signaling mechanism. Hence, the study presented here appears to refute the notion that a reduction of the C-terminal cluster is a central component of the signaling pathway. However, the high level of conservation of specific residues in group D [FeFe] hydrogenases implies a functional role of the C-terminal [4Fe-4S] cluster and the surrounding structural motifs. It remains possible that the cluster has a structural role related to a signaling process, as modification of the C-terminal [4Fe-4S] cluster had only a minor effect on the catalytic properties of *Tam*HydS. The N-terminal [4Fe-4S] clusters, on the other hand, seemed to be indispensable for the structural and functional integrity of the enzyme. Although the reduction potentials of the N- and C-terminal [4Fe-4S] clusters in *Tam*HydS were not determined, the electrostatic cluster environments provided a rationale for differently tuned [4Fe-4S] cluster potentials, resulting in a differential reduction under H_2_. For a more detailed understanding of the putative H_2_ signaling pathway of group D [FeFe] hydrogenases, future studies should focus on obtaining experimentally resolved protein structure to identify regions involved in secondary structure changes in *Tam*HydS. Furthermore, identification of the interaction partner(s) of the [FeFe] hydrogenase and elucidation of their associated protein complex are evidently critical for clarifying the signaling mechanism.

## Supplementary Material



## Notes

Ordered *Tam*HydS coding sequence primers for site-directed mutagenesis (Table S1), protein purification results (Figures S1 and S2), FeS cluster reconstitution (UV/vis spectra) (Figure S3), protein yield and Fe quantification (Table S2), purity and cofactor integration for holoenzymes (Figure S4), additional EPR spectra of apoenzyme (Figure S5), EPR spectra [3Fe-4S] cluster signal in apo-C379A and holo-C379A (Figure S6), EPR spectra and simulations of holo-C379A under H_2_ (Figure S7), EPR spectra holo-WT under H_2_–pH variation (Figure S8), specific activities for H_2_ evolution and H_2_ oxidation (Table S3), effect of H_2_ partial pressure on H_2_ oxidation currents (Figure S9), comparison of CVs at differernt pH (Figure S10), amino acid composition of N- and C-terminal domains (Table S4), Alphafold2 structure prediction and analysis (Figure S11 and S12), PDBePISA analysis of predicted homodimer interface (Table S5), list of 70 [FeFe] hydrogenases from phylogenetic group D (Table S6), and sequence conservation in N- and C-terminal domain (Figure S13) (PDF)

## †

University of Campania “Luigi Vanvitelli”, Department of Environmental, Biological and Pharmaceutical Sciences and Technologies (DiSTABiF), Caserta, Italy; Institute of Biostructures and Bioimaging (IBB-CNR), 80131 Naples, Italy

## ‡

Uppsala University, Department of Cell and Molecular Biology—Molecular Evolution, 752 37 Uppsala, Sweden; Okinawa Institute of Science and Technology—Evolution, Cell Biology, and Symbiosis Unit, 904-0495 Okinawa, Japan
