# Supplementary material for: Probing the Role of Accessory FeS Clusters in Putative Sensory Group D [FeFe] Hydrogenase: Influence of the C‑Terminal [4Fe-4S] Cluster on the Reactivity of TamHydS
Source: Biochemistry. 2026 Apr 1;65(8):1280–92. doi: 10.1021/acs.biochem.5c00784 (PMC13104023; doi:10.1021/acs.biochem.5c00784)
Supplement: Supplementary file 1 [file bi5c00784_si_001.pdf]

Supporting information

Probing the Role of Accessory FeS Clusters in Putative Sensory Group D [FeFe] Hydrogenase – Influence of the C-terminal [4Fe-4S] Cluster on Reactivity of *Tam*HydS

Conrad Schumann[1], Maximilian Böhm[1], Prasenjit Bhowmik[1, †], Ping Huang[1], Princess R. Cabotaje[1, ††], Henrik Land[1], Gustav Berggren[1, *]

[1] Uppsala University, Department of Chemistry – Ångström, Molecular Biomimetics, 751 20, Uppsala, Sweden

† Current address: University of Campania "Luigi Vanvitelli", Department of Environmental, Biological and Pharmaceutical Sciences and Technologies (DiSTABiF), Caserta, Italy; Institute of Biostructures and Bioimaging (IBB-CNR), 80131 Naples, Italy

†† Current address: Uppsala University, Department of Cell and Molecular Biology – Molecular Evolution, Uppsala, Sweden; Okinawa Institute of Science and Technology – Evolution, Cell Biology, and Symbiosis Unit, Okinawa, Japan

* Email: Gustav.berggren@kemi.uu.se

Contents

Table S1 *Tam*HydS coding sequence and primers used for site-directed mutagenesis ... 2
Figure S1 Affinity chromatography SDS-PAGE analysis of purified *Tam*HydS variants ... 3
Figure S2 Size exclusion chromatography results and final ... 4
Figure S3 FeS cluster reconstitution of *Tam*HydS WT and C379A ... 5
Table S2 Protein yield and iron per protein quantification result ... 5
Figure S4 Analysis of isolated holoenzymes regarding purity and cofactor integration ... 6
Figure S5 Additional EPR spectra of apoenzymes ... 7
Figure S6 Presence of [3Fe-4S] cluster in apo-C379A and holo-C379A ... 8
Figure S7 Spectral simulation of H_2-reduced holo-C379A and fit of holo-WT ... 9
Figure S8 FeS cluster reduction by H_2 in holo-WT at different pH ... 10
Table S3 Specific activities determind via solution assays for H_2 evolution and H_2 oxidation ... 11
Figure S9 Effect of H_2 partial pressure on H_2 oxidation currents of C379A and WT ... 12
Figure S10 Comparison of CVs of C379A and WT protein films throughout experiments ... 13
Table S4 Amio acid composition of N- and C-terminal domains with focus on charged residues ... 14
Figure S11 *In Silico* analysis of AlphaFold2 structure prediction of *Tam*HydS as homodimer ... 15
Table S5 PDBePISA (Proteins, Interfaces, Structures and Assemblies) analysis of predicted *Tam*HydS homodimer interface ... 16

Figure S12 Alphafold2 structure prediction and analysis of relative domain position 17
Table S6 List of 70 [FeFe] hydrogenases from phylogenetic group D 18
Figure S13 Sequence conservation in N- and C-terminal domain – continuous sequence 19

Table S1 *Tam*HydS coding sequence and primers used for site-directed mutagenesis

***Tam*HydS coding sequence – *E. coli* codon optimized**

ATGCTGTACTTCCACAGCGTGACCCTGGACAAGGATCGTTGCCGTGGTTGCACCAAC**TGC**A
TTAAACGTTGCCCGACCGAGGCGATCCGTGTTCGTGACGGCAAGGCGCGTATCATTAACGA
GCGTTGCATCGATTGCGGCGAATGCATTCGTGTGTGCCCGTATCACGCGAAGCTGGCGGTT
ACCGACAGCCTGGATATGATGAAAGACTTCAAATACAAAATTGCGCTGCCGGCGCCGAGC
CTGTATGGCCAGTTTCGTGATCTGACCATCAACCAAATTCTGAGCGCGCTGCTGGACGTGG
GCTTCGATGAGGTGTTTGAAGTTGCGTACGCGGCGGAGATTGTTAGCAAGTTCACCCGTGA
AGCGCTGGCGAAAGGTAACCTGAAGAAACCGGTTATTAGCAGCGCGTGCCCGGCGGTGGT
TCGTCTGGTTCAGATCCGTTTTCCGAGCCTGATTGACAACCTGCTGGATATCTGCAGCCCG
ATGGACACCGCGGCGATTCTGGCGAAGAAAGAAGCGATCAAGAAAACCGGTCTGAAAGA
GGAAGAGATCGGCGTGTTCTTTATTAGCCCGTGCGCGGCGAAGGTGACCAGCGTTAAAAA
CCCGATCGGTATTGAGAAAAGCAAGATTGACGGCGTTTTCAGCATGAAGGAAATCTACGG
CCTGATCATTCAAAAAGCGAAGACCACCGTGGTTCGTGATCTGAGCAAAGCGAGCATGAT
TGGTGTTGGTTGGGCGAACAGCGGTGGCGAGGCGTTCGGTACCTTTACCGAAAACAGCAT
CTATGTGGACGGCATTCACAACGTGGTTGATGTTCTGGAAGAGATCGAGCTGGGCAAGCT
GAACGACCTGGATTTCTTTGAAGGCCTGGCGTGCATCGGTGGCTGCATTGGTGGCCCGCTG
ACCGTGGAGAACAACTTTGTTGCGAAAAACCGTATCCGTAAGCTGACCGAAAAACTGCCG
AAGAAAGAAGAGGCGCTGTTCGACGAAGAGGAAATTGATTTTGAGGAAGTGAAGTGGAA
GAAAAAGATCGAGAAAAGCGAAGTTATGAAGCTGGACAAAGATATTAGCAAGGCGCTGG
AGATGATGAAACAGATCGACACCCAATATAAGGCGCTGCCGGGTCTGGAT**TGC**GGTAGCT
GCGGCAGCCCGACCTGCCGTGCGCTGGCGGAGGACATCGTGAAAGGCTACGCGACCGAAT
ATGACTGCATCTTCATCCTGAAGGATAAGATCAAGAACCTGAGCCAGGAGCTGAACGACC
TGGCGGGCAAGATCCCGCCGGTTCTGAGCGATGAGAAAGAATAA

***Tam*HydS variant**	Primer Sequence (5’ → 3’)
**C20A**	Forward primer: 5’-GTGGTTGCACCAAC**GCG**ATTAAAC-3’<br>Reverse primer: 5’-GTCGGGCAACGTTTAAT**CGC**GTTGG-3’
**C379A**	Forward primer: 5’-GGGTCTGGAT**GCG**GGTAGC-3’<br>Reverse primer: 5’-CGCAGCTACC**CGC**ATCCAG-3’

Mutagenesis site in bold letters and underscored.

Figure S1 Affinity chromatography SDS-PAGE analysis of purified *Tam*HydS variants

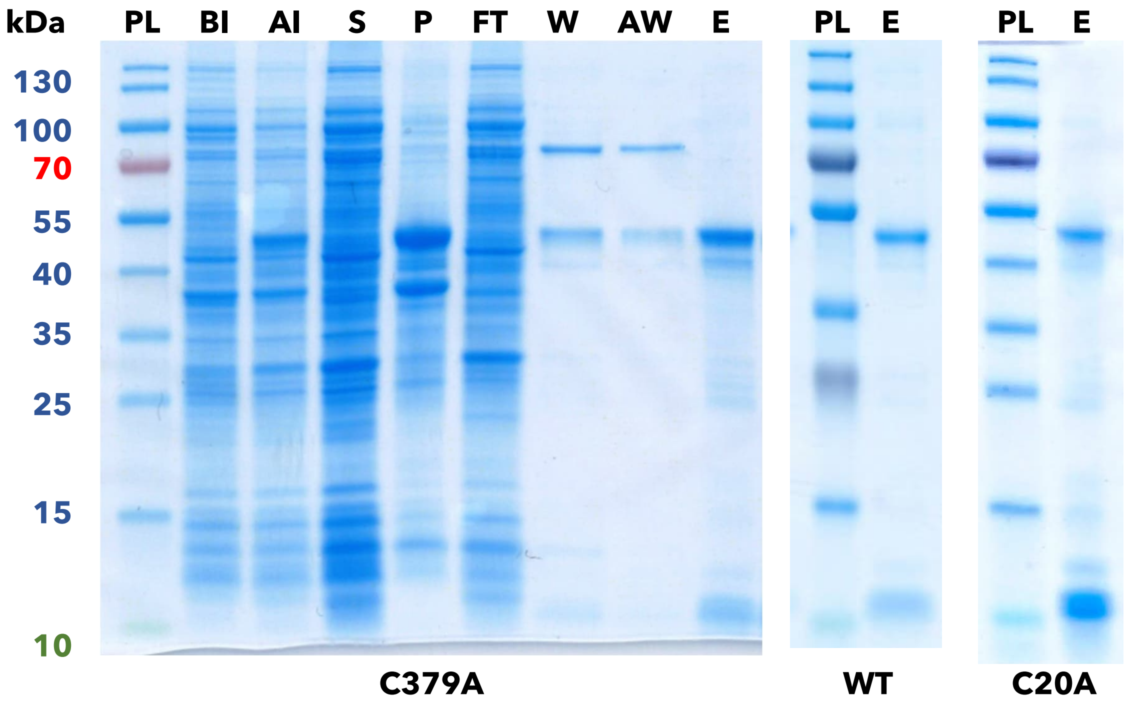

Figure S1: SDS-PAGE analysis of expressed and via affinity chromatography purified *Tam*HydS variants (WT, C20A and C379A) using StrepTrap XT columns (Cytiva) according to the manufacturer's instructions. PL: Protein standard ladder (Thermo Fisher Scientific™ PageRuler™ (Plus) Prestained Protein Ladder); BI: *E. coli* cell sample before induction; AI: after induction, S: soluble protein fraction after cell lysis; P: pellet or insoluble fraction after cell lysis; FT: flow through; W: wash; AW: ATP wash; E: elution. Higher molecular weight impurities (app. 70 kDa) could be removed through washing steps while small molecular weight impurities (app. 10 kDa) were present in the elution fractions of all three variants. For C20A the fraction of the small molecular weight impurity seems to be higher than in WT and C379A.

Figure S2 Size exclusion chromatography results

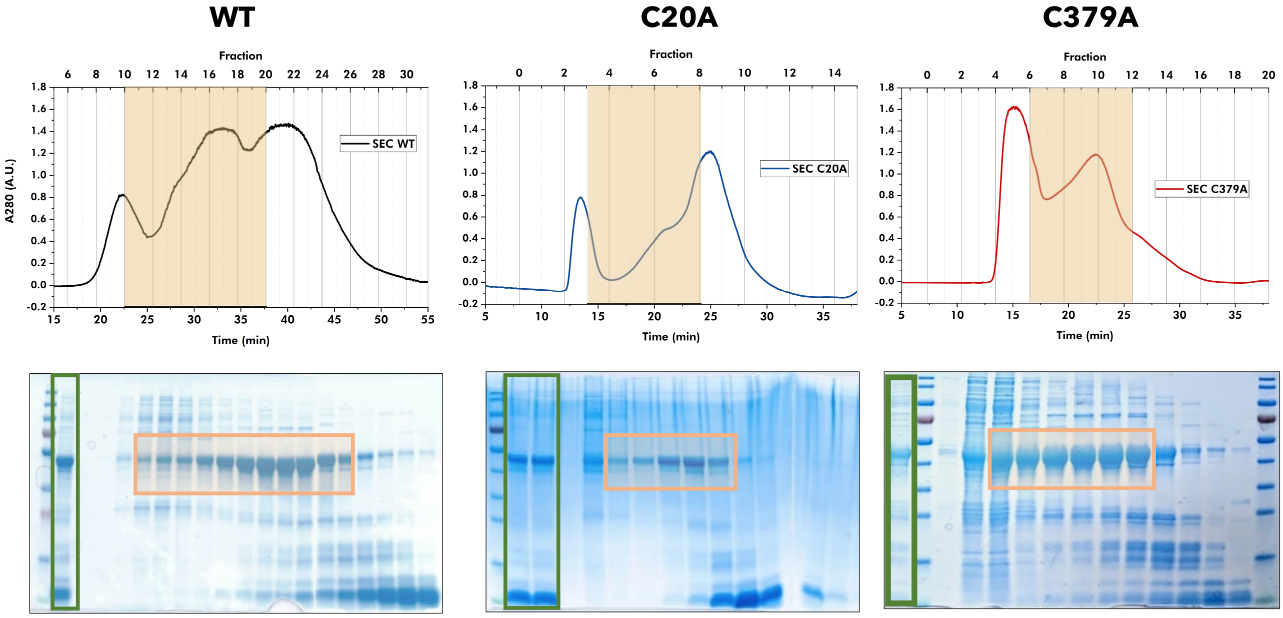

Figure S2: Size exclusion chromatograms (SEC) for WT (left), C20A (middle) and C379A (right) using a Superdex 200 Increase column (Cytiva) according to the manufacturer's instructions. SDS-PAGE analysis of fractions of the SEC runs is shown below the chromatogram. Slight variation in sample injection flow rate and fraction size caused inconsistent retention times between the runs. Orange shaded areas in the chromatograms and corresponding fractions in the SDS-PAGE highlight the combined fractions that contained the respective target proteins. Green boxes in the SDS-PAGE show the protein fraction that was loaded onto the SEC column. The initial peak in all three chromatograms is likely to stem from dimers of target proteins or protein complexes with impurities. The following peak was designated as the target protein peak based on the SDS-PAGE analysis. For C20A the chromatogram indicates a lower concentration of the target protein as the absorbance at 280 nm is lower as compared to the WT and C379A in corresponding peak of the chromatograms. These concentration differences indicate a lower protein solubility or stability for C20A.

Figure S3 FeS cluster reconstitution of *Tam*HydS WT and C379A

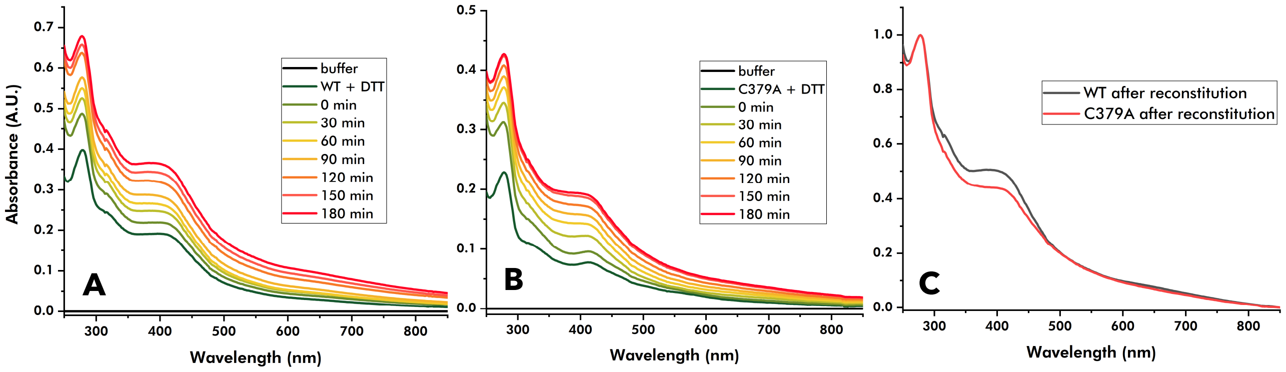

Figure S3: UV-Vis absorbance between 250 nm and 850 nm for monitoring the FeS cluster reconstitution reactions of isolated WT (A) and C379A (B) enzymes over 180 min. The reconstitution reaction of WT enzyme resulted in a steady increase of characteristic [4Fe-4S] cluster features around 280 nm and 405 nm. For the reconstitution of C379A the UV-Vis absorbance indicates a reduced occupancy of the FeS cluster sites in the beginning of the reaction as the absorbance ratio of 405 nm/280 nm is lower as compared to the WT. While the absorbance increased over the time course of the reconstitution reaction similarly as observed for the WT enzyme, it plateaued after 180 min resulting in a shoulder around 410 nm. (C) The direct comparison of the final absorbance spectra of WT and C379A, normalized at 280 nm, show clear differences in the region around 405 nm indicating differences in the iron per protein content. The spectra were collected on solution containing 50 µM enzyme in 100 mM Tris-HCl, 150 mM NaCl pH 8.0, with a 2 mm pathlength cuvette.

Table S2 Protein yield and iron per protein quantification result

	Protein yield (mg L^{-1} culture)	Fe/protein content		
		Before reconstitution	After reconstitution	After activation
**WT**	1.1	8 ± 1	14 ± 3	16 ± 3
**C20A**	0.4	12 ± 1	Not performed*	10 ± 2
**C379A**	1.4	3 ± 1	11 ± 1	14 ± 1

Table S2: After each step of the enzyme preparation the iron content per protein was monitored using a combination of protein quantification via Bradford assay and colorimetric iron quantification assays. The protein yield for the C20A was significantly lower as compared to WT and C379A further supporting the assumed differences in protein solubility and/or stability. The iron quantification indicated incomplete FeS cluster site occupancy for WT and C379A. By contrast, C20A was considered fully reconstituted with 12 Fe/protein since the disrupted N-terminal FeS cluster site was assumed to not allow FeS cluster binding. Therefore, the reconstitution reaction was not performed (*). Large standard deviations for the Fe/protein content resulted from poor precision of the Bradford assay that was calibrated with standard concentrations of BSA.

Figure S4 Analysis of isolated holoenzymes regarding purity and cofactor integration

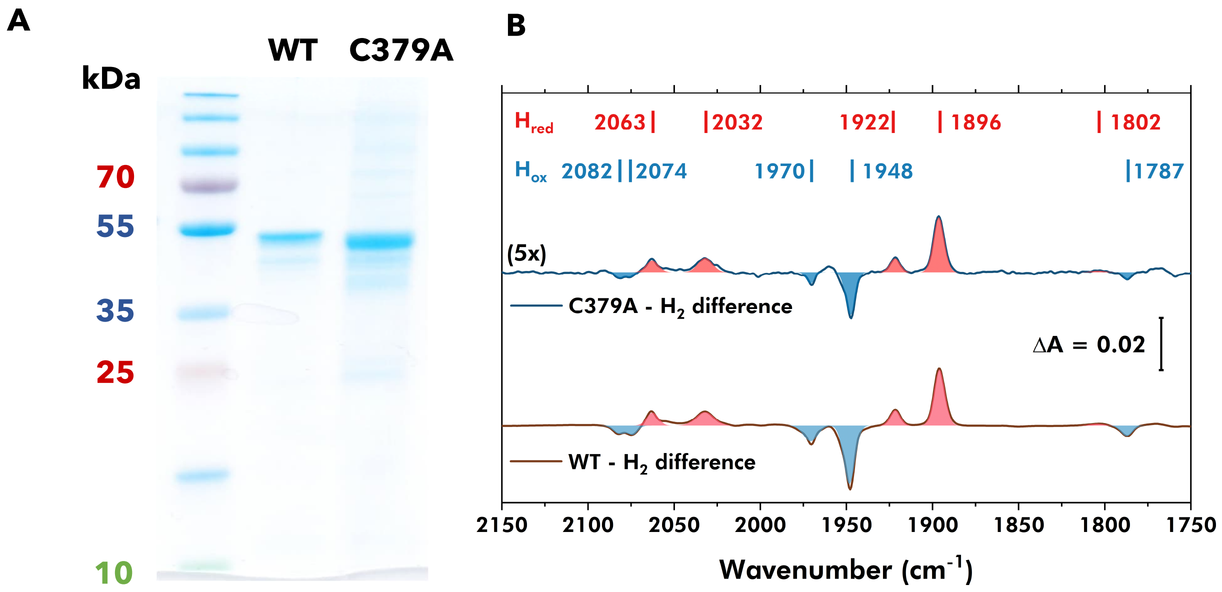

Figure S4: The isolated holoenzymes holo-WT and holo-C379A were analyzed for their final purity and the presence of the cofactor in the active site using SDS-PAGE and ATR-FTIR, respectively. (A) SDS-PAGE analysis of isolated holoenzyme of WT and C379A, loading approximately 4 and 6 µg, respectively. Both samples showed comparable purity considering different loaded amount. Thus, the small molecular weight impurity of about 12 kDa was successfully removed during the size exclusion chromatography step. (B) The cofactor integration of the prepared holoenzymes was probed measuring ATR-FTIR absorbance of a hydrated enzyme film to after exposure to N_2 followed by H_2. The resulting difference spectra revealed IR bands specific for the catalytic resting state H_{ox} and one electron reduced protonated state H_{red} (also referred to as $H_{red}H^+$) in line with the reported signatures for *Tam*HydS.[1] The fitted IR band position were virtually the same between the variant C379A and the WT with deviations of less than 1 cm^{-1}. The difference spectrum of C379A was multiplied by 5 for better comparability. The absolute spectrum of C379A indicated a mixture of H_{ox} and H_{red} in the initial state while WT was found primarily in the catalytic resting state H_{ox}. This is why C379A difference spectra showed a smaller change upon H_2 exposure.

Figure S5 Additional EPR spectra of apoenzymes

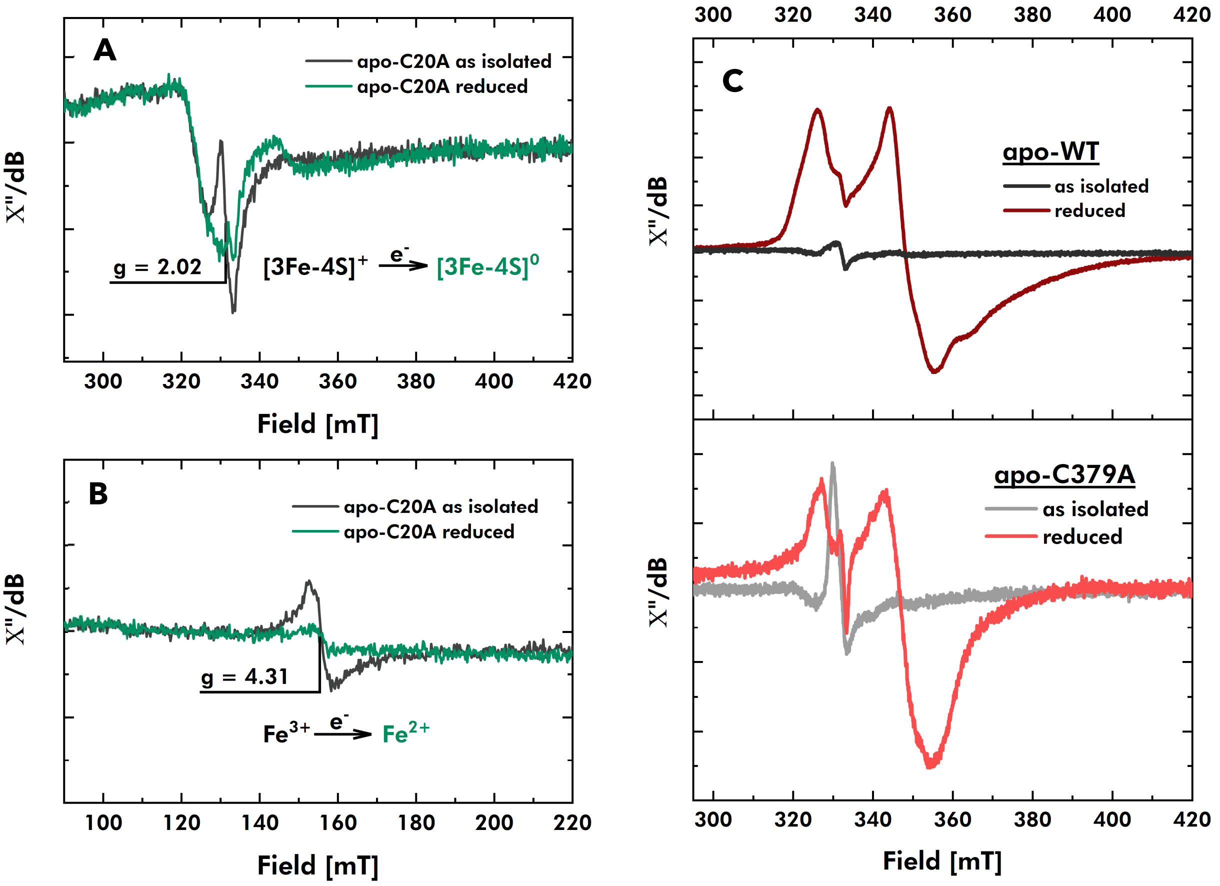

Figure S5: X-band EPR spectra of 50 µM apoenzymes (WT, C20A and C379A) in 100 mM Tris-HCl pH 8.0 comparing as isolated and NaDT reduced states. (A) and (B) show EPR spectra of isolated apo-C20A before (black) and after (green) reduction with 1 mM of NaDT. (A) Apo-C20A shows no signs of [4Fe-4S] cluster incorporation in the displayed spectral region ($g = 2.1 - 1.8$). Instead, the as-isolated samples indicates the presence of an EPR active $[3Fe\text{-}4S]^{+}$ cluster which is reduced to $[3Fe\text{-}4S]^{0}$ upon NaDT treatment causing the loss of the narrow rhombic signal centered around $g = 2.02$. (B) An analysis of the low field region around $g = 4.3$ indicated also the presence of non-specifically bound or free Fe^{3+} which was reduced to EPR-silent Fe^{2+} after NaDT treatment. (C) A direct comparison of apo-WT (dark red spectrum, top panel) and apo-C379A (red spectrum, bottom panel) spectra in the reduced states reveals the narrower rhombic signal for reduced apo-C379A as compared to apo-WT resulting from the disruption of the C-terminal [4Fe-4S] cluster. By contrast, the as isolated apo-C379A spectra show higher amounts of a [3Fe-4S] cluster with the characteristic rhombic signal centered around $g = 2.02$. The comparison of the as isolated apo-samples shows a three-fold larger $[3Fe\text{-}4S]^{+}$ signal in apo-C379A vs. apo-WT. X-band EPR spectra were collected at 10 K and 2 mW (A and B) and 80 µW (C) microwave power with microwave frequency of 9.4 GHz, modulation frequency of 100 kHz and a modulation amplitude of 1 mT.

Figure S6 Presence of [3Fe-4S] cluster in apo-C379A and holo-C379A

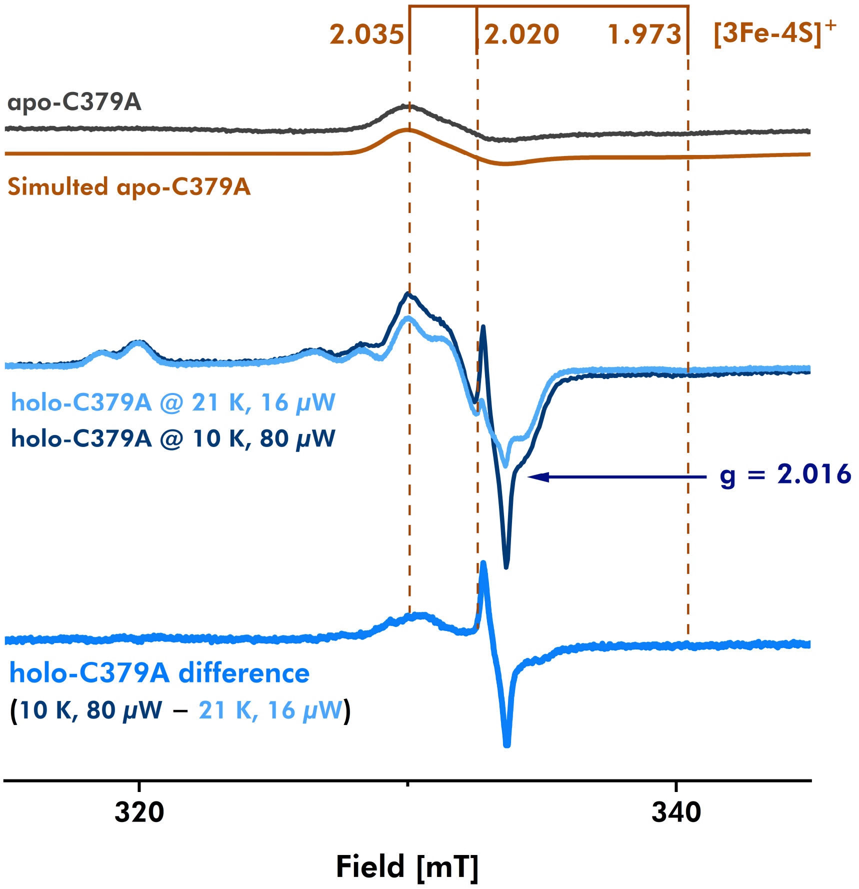

Figure S6: Comparison of apo- and holo-C379A X-band EPR spectra containing characteristic $[3Fe\text{-}4S]^{+}$ signatures. Each sample contained 50 µM enzyme in 100 mM Tris-HCl at pH 8.0. The as isolated apoenzyme of the C379A variant displayed in its EPR spectrum (grey, top) a rhombic component with g-values characteristic for $[3Fe\text{-}4S]^{+}$ ($g_{1,2,3}$ = 2.035, 2.020, 1.973). The *g*-values were determined via spectral simulations (brown, top). The holoenzyme showed at 21 K and 16 µW microwave power (light blue, middle) *Tam*HydS typical H-cluster signatures for the resting state H_{ox} with two rhombic spectral components in the *g*-range from 2.11 to 2.01. By variation of the temperature to 10 K and microwave power to 80 µW (dark blue, middle) spectral features reminiscent of the $[3Fe\text{-}4S]^{+}$ cluster and an unidentified isotropic radical-like component (*g* = 2.016) were enhanced. For the isolation of the enhanced spectral features, a difference spectrum (blue, bottom) was calculated which resembled the shape of the apoenzyme and showed an additional radical-like species. X-band EPR spectra were collected at a microwave frequency of 9.4 GHz, modulation frequency of 100 kHz and a modulation amplitude of 1 mT.

Figure S7 Spectral simulation of H_2-reduced holo-C379A and fit of holo-WT

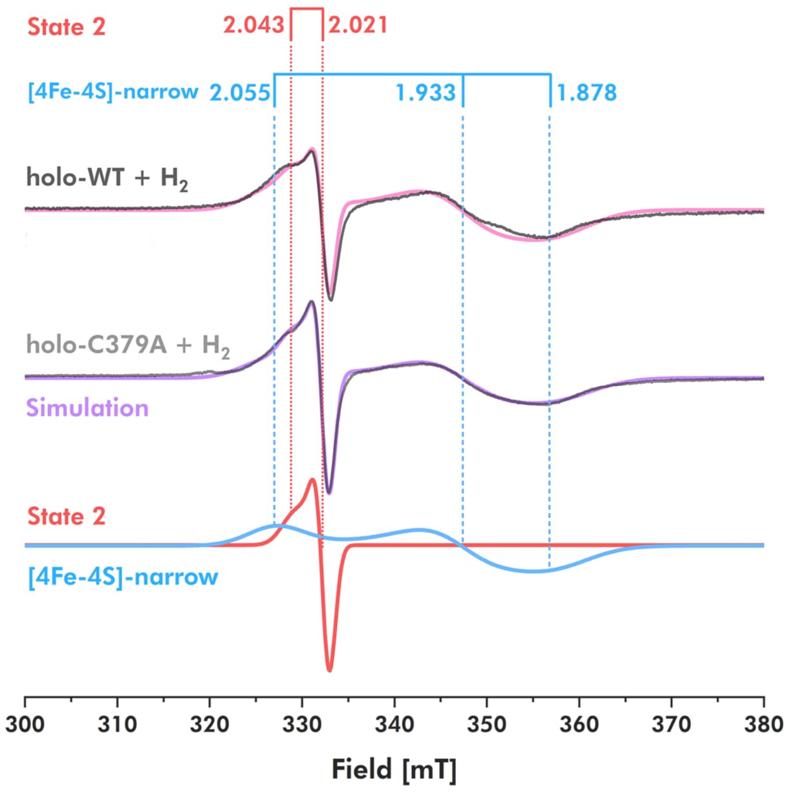

Figure S7: Comparison of X-band EPR spectra and spectral fits of H_2 reduced holo-WT and holo-C379A at pH 8.0 (50 µM enzyme). Both variants show comparable spectra with similar features but slightly different shapes. A spectral simulation for holo-C379A (purple) revealed two different spectral components, State 2 (red, $g_{||}$ = 2.043, $g_{\perp}$ = 2.021) and [4Fe-4S]-narrow (light blue, $g_{1,2,3}$ = 2.055, 1.933, 1.878). With a linear combination of the two components, it was possible to reconstruct a good fit for the H_2 reduced holo-WT spectrum (pink). This result indicates that the C-terminal [4Fe-4S] cluster in holo-WT is not reduced after H_2 exposure. X-band EPR spectra were collected at 10 K and 80 µW microwave power with a microwave frequency of 9.4 GHz, modulation frequency of 100 kHz and a modulation amplitude of 1 mT.

Figure S8 FeS cluster reduction by H_2 in holo-WT at different pH

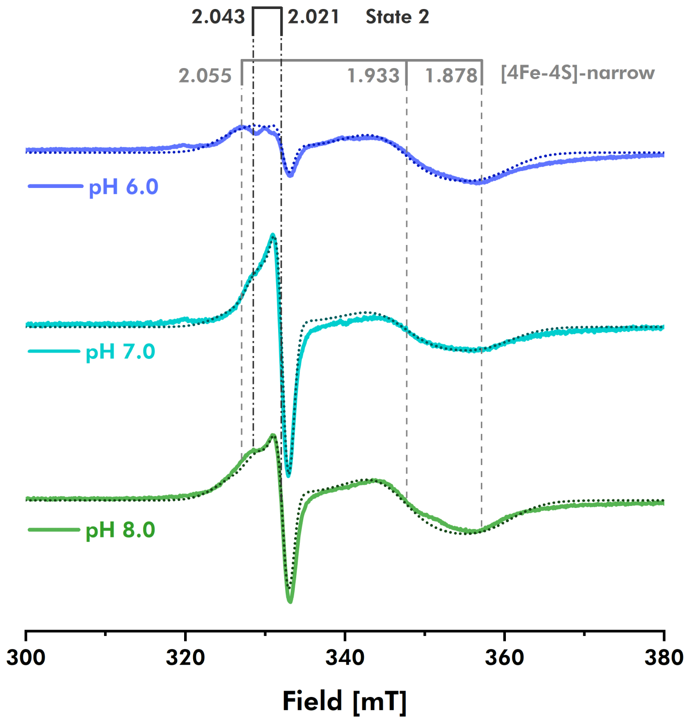

Figure S8: Normalized X-band EPR spectra of H_2 reduced *Tam*HydS holo-WT (50 µM enzyme) at pH 6.0 (blue), pH 7.0 (aquamarine) and pH 8.0 (green) show the same components in different ratios. With linear combination of the two spectral components, State 2 ($g_{||}$ = 2.043, $g_{\perp}$ = 2.021) and [4Fe-4S]-narrow ($g_{1,2,3}$ = 2.055, 1.933, 1.878) from previous spectral simulations it was possible to generate good fits for the recorded EPR spectra (dotted lines). It was not possible to obtain EPR spectra of holo-WT at a pH below pH 6.0 as the enzyme precipitated under those conditions. These results indicates that under H_2 only [4Fe-4S] clusters in the N-terminal domain are reduced while the C-terminal [4Fe-4S] cluster remains oxidized over a wider range of physiological pH. X-band EPR spectra were collected at 10 K and 80 µW microwave power with microwave frequency of 9.4 GHz, modulation frequency of 100 kHz and a modulation amplitude of 1 mT. Normalization was performed at 355 mT for a direct comparison of the characteristic [4Fe-4S]-narrow cluster signal. Samples at pH 7.0 and pH 8.0 were prepared in pH-adjusted 100 mM Tris-HCl buffer while samples at pH 6.0 were prepared in 100 mM Bis-Tris-HCl.

Table S3 Specific activities determind via solution assays for H_2 evolution and H_2 oxidation

	**H_2 evolution (MV)** $E^0 = -0.450$ V vs. SHE	**H_2 oxidation (BV)** $E^0 = -0.359$ V vs. SHE	**H_2 oxidation (MB)** $E^0 = 0.011$ V vs. SHE
**WT**	0.462 ± 0.014	1.33 ± 0.29	2.54 ± 0.34
	N=5	N=4	N=5
**C379A**	0.465 ± 0.024	1.51 ± 0.57	4.64 ±v0.80
	N=6	N=6	N=10

Table S3: Tabulated specific activities (U/mg) for H_2-evolution (H^+-reduction) and H_2-oxidation using gas chromatography and colorimetric assays, respectively, 1 U = 1 µmol H_2 produced or oxidized per min. H_2-evolution activities were measured at pH 6.8 and 30°C using 10 mM methyl viologen (MV, $E^0 = -0.450$ V vs. SHE) as redox mediator and 100 mM NaDT as sacrificial electron donor. H_2-oxidation activities were measured with 500 µM benzyl viologen (BV, $E^0 = -0.359$ V vs. SHE) or 50 µM methylene blue (MB, $E^0 = 0.011$ V vs. SHE) in H_2-saturated phosphate buffer at pH 6.8 and 25 °C. [2-4] Values presented as mean and standard deviation with the number of technical replicates indicated below.

Figure S9 Effect of H_2 partial pressure on H_2 oxidation currents of C379A and WT

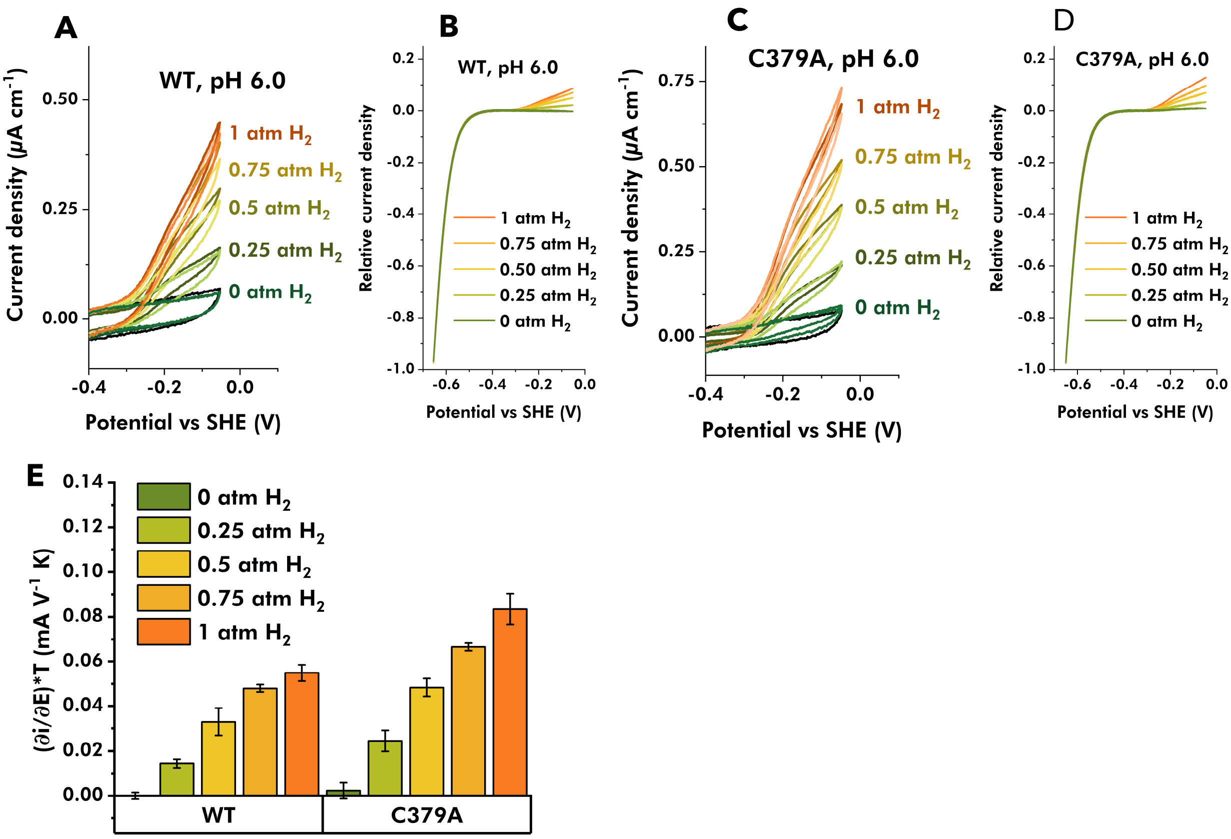

Figure S9: Analysis of protein film voltammetry (PFV) comparing the *Tam*HydS variant C379A with WT with varying H_2 concentrations in the headspace of the electrochemical cell from 1 atm to 0 atm H_2. Enzymes were immobilized on individual rotating disc edge-plane pyrolytic graphite electrodes (PGE) in the presence of polymyxin B sulfate. CVs for WT (A) and C379A (C) were repeatedly recorded at pH 6.0 with varying H_2 concentrations for each enzyme, represented by various traces for the different H_2 concentrations. CVs collected for the enzymes films of (B) WT and (D) C379A were then normalized to the reductive peak current for better comparability. (E) The slopes of the CVs (A and B) were analyzed to estimate the steady state limiting current for the H_2-oxidation. Data is presented as the average of the resulting slopes with the error bars representing the standard deviation between repeats. Based on the analysis it can be said that both enzymes showed a steady increase in the oxidation currents with increasing H_2 partial pressure. The H_2 oxidation currents for C379A were higher compared to the WT.

Figure S10 Comparison of CVs of C379A and WT protein films throughout experiments

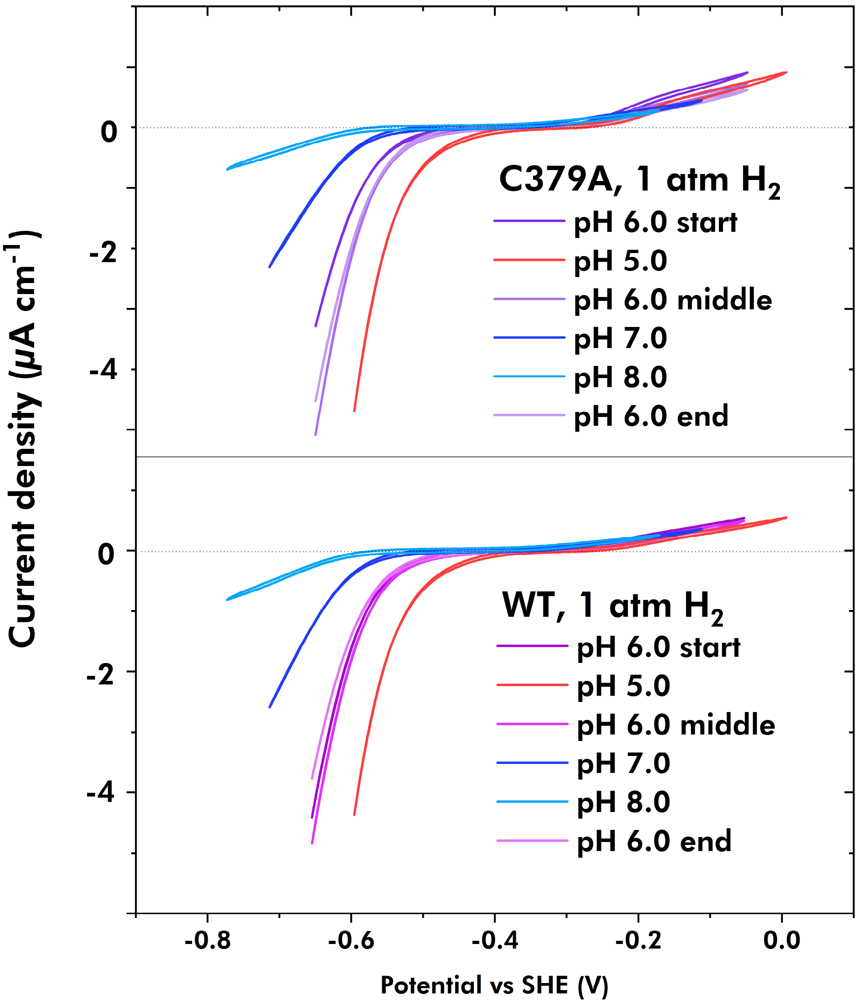

Figure S10: Comparison of CVs generated with protein films using *Tam*HydS variant C379A and WT. Enzymes were immobilized on individual rotating disc edge-plane pyrolytic graphite electrodes (PGE) in the presence of polymyxin B sulfate. Film stability and reproducibility of measured currents was verified by returning to the initial conditions (pH 6.0, 1 atm H_2) in the middle (after 2 h) and at the end of the experiments (after 4h). Only minor deviations in the measured currents were observed for both films. For C379A the initial CV showed lower reductive current, while the oxidative currents were only slightly higher than in the repeated measurements. The measurement repeats (pH 6.0) on the other hand resulted in highly similar CV traces for C379A. This observation indicates that the enzyme was reductively activated during the first measurement at pH 6.0.

Table S4 Amio acid composition of N- and C-terminal domains with focus on charged residues

	***Tam*HydS**		
	**N-terminal domain**	**C-terminal domain**	
	Complete domain	Complete domain	~15Å from [4Fe-4S]
**Residue (total)**	1 – 60 (60)	349 – 435 (87)	366 – 410 (45)
**Theoretical pI**	8.90	4.73	4.33
**Amino acid composition:**			
Ala (A)	6.70 %	6.90 %	8.90 %
**Arg (R)**	**13.30 %**	**1.10 %**	**2.20 %**
Asn (N)	3.30 %	2.30 %	0.00 %
**Asp (D)**	**6.70 %**	**10.30 %**	**11.10 %**
Cys (C)	13.30 %	4.60 %	8.90 %
Gln (Q)	0.00 %	3.40 %	4.40 %
**Glu (E)**	**5.00 %**	**8.00 %**	**4.40 %**
Gly (G)	5.00 %	5.70 %	8.90 %
His (H)	3.30 %	0.00 %	0.00 %
Ile (I)	10.00 %	8.00 %	8.90 %
Leu (L)	5.00 %	11.50 %	8.90 %
**Lys (K)**	**6.70 %**	**12.60 %**	**6.70 %**
Met (M)	1.70 %	3.40 %	0.00 %
Phe (F)	1.70 %	1.10 %	2.20 %
Pro (P)	3.30 %	4.60 %	4.40 %
Ser (S)	1.70 %	5.70 %	4.40 %
Thr (T)	5.00 %	3.40 %	6.70 %
Trp (W)	0.00 %	0.00 %	0.00 %
Tyr (Y)	3.30 %	3.40 %	6.70 %
Val (V)	5.00 %	3.40 %	2.20%
**Negative charge**	**7 (11.7 %)**	**16 (18.3 %)**	**7 (15.5 %)**
**Positive charge**	**12 (20 %)**	**12 (13.7 %)**	**4 (8.9 %)**

Table S4: The amino acid composition of the N- and C-terminal domains was analyzed using the ProtParam tool on the ExPasy Server.[5] In the N-terminal domain (residue 1 - 60), 20 % of the residues are positively charged residues (Lys or Arg) while negatively charged (Asp and Glu) residues make up 11.7 %. Conversely, the C-terminal domain is composed of 13.7 % positively charged and 18.3% negatively charged residues. In the vicinity of the C-terminal [4Fe-4S] cluster (radius < 15 Å) the fraction of positively charged residues was even lower 8.9 %. These local differences in the amino acid composition are also reflected in the theoretical isoelectric points (pI) of 8.9 and 4.7 for the N- and C-terminal domains, respectively.

Figure S11 *In Silico* analysis of AlphaFold2 structure prediction of *Tam*HydS as homodimer

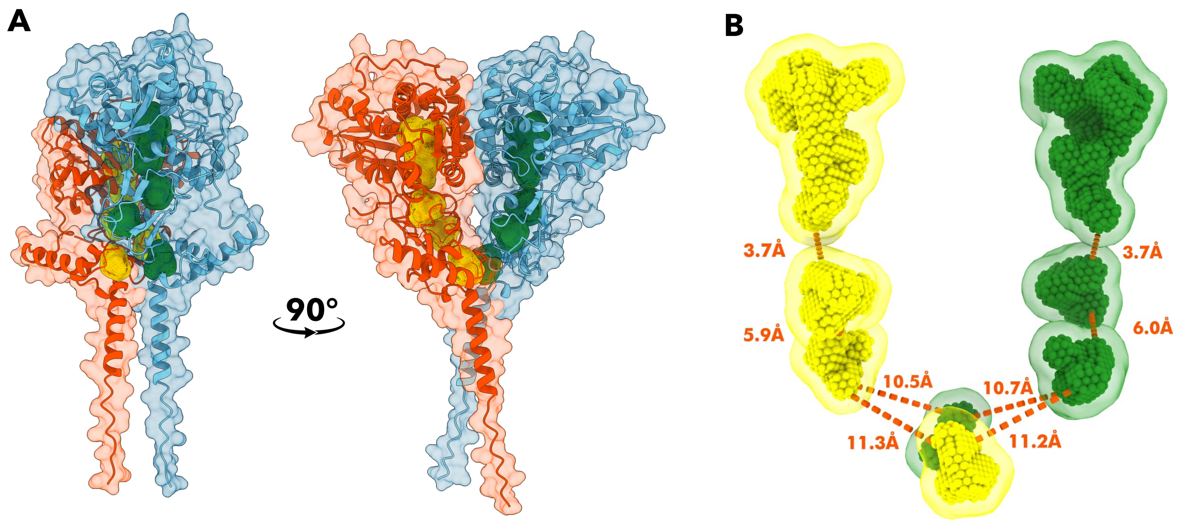

Figure S11: AlphaFold2 (AF2) structure prediction of a TamHydS homodimer including distances between cofactor binding sites in the predicted cavities. (A) AlphaFold2 predicts the interactions between two *Tam*HydS protomers (red and blue cartoon with transparent surface) in which the protomer structures remain largely unchanged when compared to the isolated monomer prediction. Using pyKVfinder[6] in ChimeraX[7], the [4Fe-4S] cluster binding sites were modeled in the predicted protein scaffold cavities (yellow volumes in red subunit and green volumes in blue subunit). (B) The enlarged and isolated view of the predicted cofactor binding sites reveals small distances between the N-terminal accessory [4Fe-4S] cluster sites and the H-cluster site (3.7 - 6.0 Å). By contrast the distance between the N- and C-terminal accessory [4Fe-4S] cluster sites within a protomer is larger with (10.7 - 11.3 Å). The distance between N- and C-terminal accessory [4Fe-4S] cluster sites from different protomers is also to predicted with similar distances (10.5 - 11.2 Å). The predicted dimer shows therefore no clear differences for the electron transfer distance to the C-terminal accessory [4Fe-4S] cluster. However, electron tunneling remains possible for the predicted distances to the C-terminal [4Fe-4S] cluster site.

Table S5 PDBePISA (Proteins, Interfaces, Structures and Assemblies) analysis of predicted *Tam*HydS homodimer interface

	Structure 1		Structure 2	
**Subunit**	TamHydS - Chain A		TamHydS - Chain B	
**Number of atoms**				
interface	176	5.2%	176	5.2%
surface	2022	59.9%	2029	60.1%
total	3376	100.0%	3376	100.0%
**Number of residues**				
interface	55	12.6%	54	12.4%
surface	402	92.4%	400	92.0%
total	435	100.0%	435	100.0%
**Solvent-accessible area, Å**				
interface	1863.7	8.1%	1853.9	8.1%
total	22991.1	100.0%	22994.2	100.0%
**Solvation energy, kcal/mol**				
isolated structure	-432.7	100.0%	-434.3	100.0%
gain on complex formation	-1.6	0.4%	-1.1	0.3%
average gain	-3.2	0.7%	-3.0	0.7%
P-value	0.640		0.667	
**Complex Formation Significance Score (CSS) is 0.00 → no relevance in complex formation**				

Table S5: *Tam*HydS homodimer was analyzed using the PDBePISA online tool in order to evaluate the predicted interaction. A low gain in calculated solvation energy upon complex formation indicated low relevance for the interaction in promotion of a complex formation. Hence, *Tam*HydS structures and domain interactions were only analyzed in the context of the predicted monomer structure.

Figure S12 Alphafold2 structure prediction and analysis of relative domain position

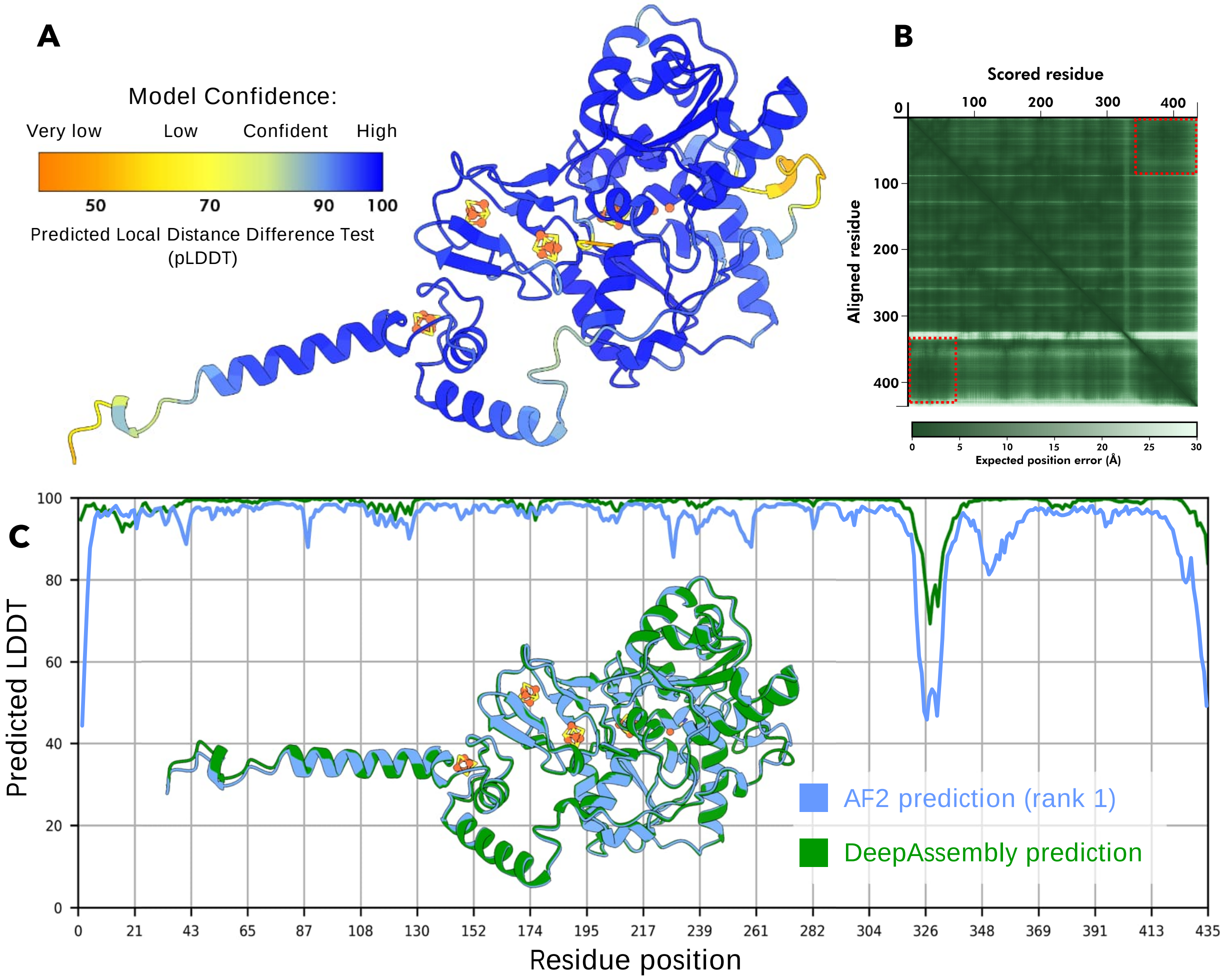

Figure S12: Structure predictions by AlphaFold2 (AF2) and DeepAssembly were used as basis for structural analysis of *Tam*HydS. [8, 9] N-terminal [4Fe-4S] clusters were modeled into the predicted protein structure by aligning the X-ray crystal structure of *Cp*I (PDB:4XDC) with *Tam*HydS while the C-terminal cluster was fitted manually, considering Fe-S-bond distances of approximately 2.2 Å. (A) The *Tam*HydS structure displayed as ribbon is colored according to the per-residue Predicted Local Distance Difference Test (pLDDT) confidence score, following the standard AF2 color scheme. The AF2 prediction resulted in a model with a mostly high pLDDT values (global pLDDT = 94.2) which indicates a high confidence of the structural prediction. Regions of low model confidence are the N- and C-termini as well as a loop region between the H-domain and C-terminal domain (residues 323-334). (B) The Predicted Aligned Error (PAE) plot for *Tam*HydS displays the expected position error (in Ångstrom, Å) for every residue and carries information about the certainty of the relative domain arrangement. Low expected position errors in the regions for the relative positions of the C- and N-terminal domain suggest a relative rigid structure with interdomain interactions (red dotted boxes). (C) The comparison of the AF2 model (blue) with the DeepAssembly structure prediction (green) reveals only negligeable structural differences, reflected in a RMSD of 0.5 Å after alignment. Additionally, the DeepAssembly results in a higher pLDDT score in most positions supporting the notion of a highly confident structural prediction with a rigid interaction between the N- and C-terminal domains of *Tam*HydS.

Table S6 List of 70 [FeFe] hydrogenases from phylogenetic group D

Accession Number	Organism Name	Accession Number	Organism Name
WP_014163756.1	*Thermovirga lienii*	WP_027355352.1	*Desulfofundulus thermocisternus*
WP_007656801.1	*Parabacteroides goldsteinii*	WP_027717491.1	*Desulfovirgula thermocuniculi*
WP_008157723.1	*Parabacteroides johnsonii*	WP_018084276.1	*Desulfurispora thermophila*
WP_028729380.1	*Parabacteroides*	WP_027364040.1	*Desulfallas alcoholivorax*
WP_005643245.1	*Parabacteroides merdae*	WP_006521135.1	*Desulfallas gibsoniae*
WP_026475068.1	*Alkaliflexus imshenetskii*	WP_028308072.1	*Desulfitibacter alkalitolerans*
WP_016777702.1	*Anaerophaga thermohalophila*	WP_021167205.1	*Sporomusa ovata*
WP_010665314.1	*Marinilabilia salmonicolor*	WP_007288806.1	*Thermosinus carboxydivorans*
WP_013610546.1	*Odoribacter splanchnicus*	WP_013778946.1	*Tepidanaerobacter acetatoxydans*
WP_019541391.1	*Proteiniphilum acetatigenes*	WP_015907773.1	*Caldicellulosiruptor bescii*
WP_008909343.1	*Caloramator australicus*	WP_013403323.1	*Caldicellulosiruptor hydrothermalis*
WP_018660455.1	*Thermobrachium celere*	WP_013290556.1	*Caldicellulosiruptor obsidiansis*
WP_013657399.1	*Cellulosilyticum lentocellum*	**WP_418365830.1**	***Thermoanaerobacter mathranii***
WP_015327627.1	*Halobacteroides halobius*	WP_013780872.1	*Mahella australiensis*
WP_027338883.1	*Halonatronum saccharophilum*	WP_026893765.1	*Clostridiisalibacter paucivorans*
WP_018247867.1	*Orenia marismortui*	WP_010251919.1	*Hungateiclostridium cellulolyticum*
WP_013406358.1	*Halanaerobium hydrogeniformans*	WP_014254572.1	*Hungateiclostridium clariflavum*
WP_012939176.1	*Acidaminococcus fermentans*	WP_003512576.1	*Hungateiclostridium thermocellum*
WP_009145644.1	*Phascolarctobacterium succinatutens*	WP_015358348.1	*Thermoclostridium stercorarium*
WP_012446583.1	*Natranaerobius thermophilus*	WP_027629168.1	*Ruminiclostridium cellobioparum*
WP_002572006.1	*Enterocloster bolteae*	WP_004626210.1	*Ruminiclostridium cellobioparum*
WP_027642172.1	*Enterocloster clostridioformis*	WP_026894022.1	*Clostridiisalibacter paucivorans*
WP_007862856.1	*Clostridiales*	WP_018962522.1	*Coprothermobacter platensis*
WP_006778972.1	*Hungatella hathewayi*	WP_012543804.1	*Coprothermobacter proteolyticus*
WP_013273980.1	*Lacrimispora saccharolytica*	WP_014758976.1	*Thermoanaerobacterium aotearoense*
WP_015513457.1	*Coprococcus catus*	WP_013788354.1	*Thermoanaerobacterium xylanolyticum*
WP_004608279.1	*[Clostridium] scindens*	WP_013297601.1	*Thermoanaerobacterium thermosaccharolyticum*
WP_021739962.1	*Eubacterium ramulus*	WP_026485859.1	*Caldanaerobius polysaccharolyticus*
WP_003534146.1	*[Clostridium] leptum*	WP_009609830.1	*Caldanaerobacter subterraneus*
WP_012064286.1	*Alkaliphilus metalliredigens*	WP_028991495.1	*Thermoanaerobacter thermocopriae*
WP_006573282.1	*Pseudoflavonifractor capillosus*	WP_014063294.1	*Thermoanaerobacter wiegelii*
WP_026393511.1	*Acetobacterium dehalogenans*	WP_012994906.1	*Thermoanaerobacter italicus*
WP_014357055.1	*Acetobacterium woodii*	WP_004401673.1	*Thermoanaerobacter thermohydrosulfuricus*
WP_013379176.1	*Clostridiales*	WP_003871510.1	*Thermoanaerobacter ethanolicus*
WP_013823363.1	*Desulfofundulus sp.* TPOSR	WP_006570151.1	*Thermoanaerobacter siderophilus*

Table S6: List of other [FeFe] hydrogenases from phylogenetic group D with accession numbers and organisms including *Tam*HydS (WP_418365830.1).

Figure S13 Sequence conservation in N- and C-terminal domain – continuous sequence

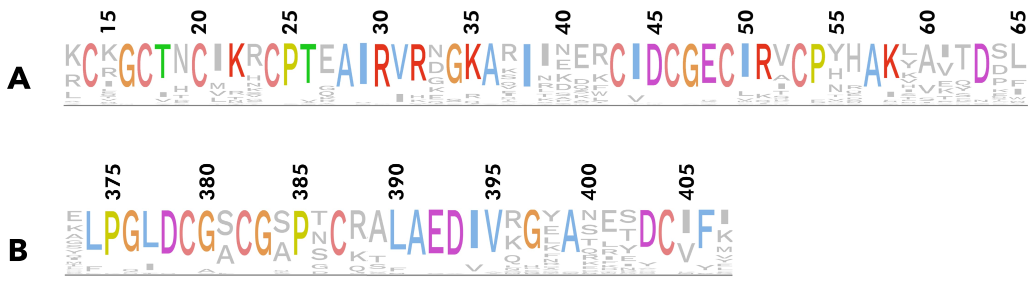

Figure S13: Sequence conservation across group D [FeFe] hydrogenases in (A) the N-terminal and (B) C-terminal [4Fe-4S] cluster binding domains and the direct sequence environment of the cluster binding cysteines. The sequence logo is based on 70 [FeFe] hydrogenases from the phylogenetic group D which were aligned using ClustalOmega. Colored residues are considered highly conserved with a residue conservation of 75 % and higher. (A) The sequence conservation in the N-terminal domain shows, in addition to the highly conserved cysteines also a higher conservation of positively charged lysine and arginine residues compared to the negatively charged aspartate and glutamate residues. (B) Contrarily, the C-terminal domain displays no conservation of positively charged residues but instead highly conserved aspartate and glutamate residues. The residues D378, E392 and D393 show even a 100 % residue conservation which indicates important functional roles for these residues.

References

(1) Land, H.; Sekretareva, A.; Huang, P.; Redman, H. J.; Németh, B.; Polidori, N.; Mészáros, L. S.; Senger, M.; Stripp, S. T.; Berggren, G. Characterization of a putative sensory [FeFe]-hydrogenase provides new insight into the role of the active site architecture. *Chemical Science* **2020**, *11* (47), 12789-12801. DOI: 10.1039/d0sc03319g.
(2) Van Den Heuvel, R. H. H.; Fraaije, M. W.; Van Berkel, W. J. H. Redox Properties of Vanillyl-Alcohol Oxidase. Elsevier, 2002; pp 177-186.
(3) Wardman, P. The Reduction Potential of Benzyl Viologen: An Important Reference Compound for Oxidant/Radical Redox Couples. *Free Radical Research Communications* **1991**, *14* (1), 57-67. DOI: 10.3109/10715769109088942.
(4) Impert, O.; Katafias, A.; Kita, P.; Mills, A.; Pietkiewicz-Graczyk, A.; Wrzeszcz, G. Kinetics and mechanism of a fast leuco-Methylene Blue oxidation by copper(ii)–halide species in acidic aqueous media. *Dalton Transactions* **2003**, (3), 348-353. DOI: 10.1039/b205786g.
(5) Gasteiger, E. ExPASy: the proteomics server for in-depth protein knowledge and analysis. *Nucleic Acids Research* **2003**, *31* (13), 3784-3788. DOI: 10.1093/nar/gkg563.
(6) Guerra, J. V. D. S.; Ribeiro-Filho, H. V.; Jara, G. E.; Bortot, L. O.; Pereira, J. G. D. C.; Lopes-De-Oliveira, P. S. pyKVFinder: an efficient and integrable Python package for biomolecular cavity detection and characterization in data science. *BMC Bioinformatics* **2021**, *22* (1). DOI: 10.1186/s12859-021-04519-4.
(7) Goddard, T. D.; Huang, C. C.; Meng, E. C.; Pettersen, E. F.; Couch, G. S.; Morris, J. H.; Ferrin, T. E. UCSF ChimeraX: Meeting modern challenges in visualization and analysis. *Protein Science* **2018**, *27* (1), 14-25. DOI: 10.1002/pro.3235.

(8) Xia, Y.; Zhao, K.; Liu, D.; Zhou, X.; Zhang, G. Multi-domain and complex protein structure prediction using inter-domain interactions from deep learning. *Communications Biology* **2023**, *6* (1). DOI: 10.1038/s42003-023-05610-7.
(9) Jumper, J.; Evans, R.; Pritzel, A.; Green, T.; Figurnov, M.; Ronneberger, O.; Tunyasuvunakool, K.; Bates, R.; Žídek, A.; Potapenko, A.; et al. Highly accurate protein structure prediction with AlphaFold. *Nature* **2021**, *596* (7873), 583-589. DOI: 10.1038/s41586-021-03819-2.
